# Biogeography, succession, and origin of the chicken intestinal mycobiome

**DOI:** 10.1186/s40168-022-01252-9

**Published:** 2022-04-01

**Authors:** Kelsy Robinson, Qing Yang, Sydney Stewart, Melanie A. Whitmore, Guolong Zhang

**Affiliations:** 1 Department of Animal and Food Sciences, Oklahoma State University, Stillwater, Oklahoma USA; 2 Present Address: Poultry Research Unit, USDA–Agricultural Research Service, Mississippi State, MS USA; 3 Present Address: Safety and Security Division, Institute for Public Research, CNA, Arlington, VA USA

**Keywords:** Mycobiome, Microbiome, Fungal community, Poultry

## Abstract

**Background:**

Extensive work has been accomplished to characterize the intestinal bacterial community, known as the microbiota, and its association with host health and disease. However, very little is known about the spatiotemporal development and the origin of a minor intestinal fungal community, known as the mycobiota, in humans and animals, particularly in avian species.

**Results:**

In this study, we comprehensively characterized the biogeography and succession of the gastrointestinal (GI) mycobiota of broiler chickens and further revealed the fungal sources that are responsible for initial and long-term establishment of the mycobiota in the GI tract. Using Illumina sequencing of the internal transcribed spacer 2 (ITS2) region of fungal rRNA genes, we detected significant spatial and temporal differences in the mycobiota along the GI tract. In contrary to the microbiota, the mycobiota was more diverse in the upper than the lower GI tract with no apparent trend of succession up to 42 days of age. The intestinal mycobiota was dominated by the phyla Ascomycota and Basidiomycota with *Gibberella*, *Aspergillus*, and *Candida* being the most abundant genera. Although the chicken mycobiota was highly dynamic, *Fusarium pseudonygamai* was dominant throughout the GI tract regardless of age in this study. The core chicken mycobiome consisted of 26 fungal taxa accounting for greater than 85% of the fungal population in each GI location. However, we observed high variations of the intestinal mycobiota among different studies. We also showed that the total fungal population varied greatly from 1.0 × 10^4^ to 1.1 × 10^6^ /g digesta along the GI tract and only accounted for less than 0.06% of the bacteria in day-42 broilers. Finally, we revealed that the mycobiota from the hatchery environment was responsible for initial colonization in the GI tract of newly hatched chickens, but was quickly replaced by the fungi in the diet within 3 days.

**Conclusions:**

Relative to the intestinal microbiota that consists of trillions of bacteria in hundreds of different species and becomes relatively stabilized as animals age, the chicken intestinal mycobiota is a minor microbial community that is temporally dynamic with limited diversity and no obvious pattern of successive changes. However, similar to the microbiota, the chicken mycobiota is spatially different along the GI tract, although it is more diverse in the upper than the lower GI tract. Dietary fungi are the major source of the intestinal mycobiota in growing chickens.

Video abstract

**Supplementary Information:**

The online version contains supplementary material available at 10.1186/s40168-022-01252-9.

## Background

The gastrointestinal (GI) tract of humans and animals is inhabited by a large diverse community of bacteria, known as the microbiota, and other smaller and generally less diverse communities of fungi, viruses, archaea, and protozoa [1, 2]. The advent of next-generation sequencing has vastly expanded our knowledge on the intestinal microbiota in humans, mice, livestock animals, and avian species [3–6]. Changes in the bacterial community are well known to be associated with a broad range of intestinal and extra-intestinal diseases [1, 2]. Few studies have investigated smaller microbial populations in the GI tract, although the role of the fungal community, known as the mycobiota, in host health and disease is being actively explored [7–10].

In humans, the mycobiota is estimated to account for approximately 0.02% of the intestinal mucosa-associated microbiota and 0.03% of the fecal microbiota [11]. Investigations into the oral and intestinal fungal residents have revealed the phyla Ascomycota and Basidiomycota to be dominant, while the genera commonly include *Saccharomyces*, *Candida*, *Aspergillus*, *Cladosporium*, *Penicillium*, *Wallemia*, *Malassezia*, *Aureobasidium*, and *Epicoccum* [12]. Fungal dysbiosis is linked to several diseases such as inflammatory bowel disease, allergic airway disease, atopic dermatitis, and alcoholic liver disease [7–10]. In mice, Ascomycota and Basidiomycota are also identified as the major phyla, with *Candida*, *Saccharomyces*, *Trichosporon*, *Aspergillus*, *Penicillium*, *Wickerhamomyces*, *Cladosporium*, and *Fusarium* being among the most abundant genera [13–16].

In livestock animals, the mycobiome research has primarily focused on ruminant animals with special attention given to anaerobic fungi in the phylum Neocallimastigomycota [6, 17]. In pigs, *Kazachstania* is the most dominant along with *Hyphopichia* and *Wallemia* [18, 19]. The mycobiome studies are even more limited in avian species. Most are culture-based and restricted only to the cecum [20–25]. *Candida* or *Aspergillus* are predominant in the chicken cecum in culture-dependent studies. A culture-independent pyrosequencing study only detected two *Cladosporium* species in the cecum of broilers fed unmedicated diet [20]. In turkeys, ITS2 sequencing of the ileum content recently found *Sarocladium kiliense* to be the most abundant, while *Candida* was only observed in probiotic- and antibiotic-treated chickens [26]. Previous work in our lab used Illumina sequencing to investigate the intestinal mycobiome on day-28 chickens. We revealed *Microascus* to be the predominant genus and observed an obvious difference in the cecal mycobiome composition between day-14 and day-28 chickens [27]. However, a detailed in-depth analysis of the spatiotemporal development of the intestinal mycobiome of an animal species is lacking. In addition, the fungal sources that are responsible for initial and long-term establishment of the intestinal mycobiota remains unknown.

Using chickens as an animal model, we sought to comprehensively investigate the biogeography of the mycobiota along the GI tract in this study. We also systematically characterized successional changes of the chicken mycobiome in four different intestinal segments (the duodenum, jejunum, ileum, and cecum) at seven different time points (days 3, 7, 14, 21, 27, 35, and 42) throughout an entire production cycle of broilers. Furthermore, we attempted to determine the origin of the intestinal mycobiota in chickens by dissecting the influence of the mycobiota in the hatchery, diets, and housing environment on the intestinal mycobiota during the first week of life.

## Material and methods

### Animal trials and sample collection

Animal trials were conducted in accordance with the Institutional Animal Care and Use Committee of Oklahoma State University under protocol number AG-173. For fungal biogeography and succession studies, 120 day-of-hatch male Cobb broiler chicks were obtained from Cobb-Vantress Hatchery (Siloam Springs, AR) and randomly assigned to 12 floor cages with 10 chickens per cage. Chickens were raised for 6 weeks under standard management with ad libitum access to feed and tap water for the entire duration of the trial. Non-medicated soybean-corn-based starter, grower, and finisher diets were formulated to meet or exceed the National Research Council (NRC) recommendations. Feed was switched from starter to grower and grower to finisher on days 14 and 27, respectively. Chickens were raised on floor pens with fresh dry pinewood shavings in an environmentally controlled room with temperature starting at 33 °C and decreasing 3 °C every week. Lighting for this trial included the light-to-dark ratio of 23:1 from day 0 to 7 and 16:8 from day 8 to 42. On days 3, 7, 14, 21, 27, 35, and 42, twelve chickens from 12 different cages with one per cage were euthanized via CO_2_ asphyxiation. Approximately 0.20–0.3 g of the digesta was aseptically collected from the mid-duodenum, mid-jejunum, mid-ileum, and left cecum of each animal by gentle squeeze into preweighed ZR BashingBead™ Lysis Tubes (Zymo Research, Irvine, CA). Sample collection on days 14 and 27 was performed prior to feed transitions. Additionally, the crop, ventriculus, and colon were cut open and 0.20–0.3 g of the digesta was aseptically collected into preweighed ZR BashingBead™ Lysis Tubes from 12 chickens in 12 cages on day 42 for characterization of spatial differences in the chicken mycobiome. All digesta sample tubes were snap frozen in liquid nitrogen and stored at – 80 °C till processing.

Another 60 chickens from the same batch were used to identify the source of the mycobiota that initially colonizes the GI tract of young chicks. Different types of environmental samples were collected from the poultry room 2 h prior to the arrival of chicks. An airborne dust sample was collected by placing three petri dishes approximately 1 m above the ground for 30 min, followed by rinsing with 4 mL of sterile PBS per dish and collection into a 12-mL conical tube for centrifugation and DNA isolation. A 10-mL tap water sample was collected in a 12-mL conical tube from the waterline supplying bird cages, while the wire mesh pens were sampled by swabbing all four sides with two swabs pre-soaked in sterile PBS. Two 5-g feed samples and two 10-g wood shaving samples were placed in preweighed Ziploc bags that had been pre-rinsed with sterile PBS.

Upon arrival in a single enclosed cardboard box at the poultry room, 40 chicks were allotted to a large floor pen with free access to feed and water, subjected to the same management and feeding procedures as described in the biogeography and succession studies, and used for collection of the intestinal contents on days 3 and 7, while the other 20 chicks were immediately euthanized via CO_2_ asphyxiation for collection of feather and the digesta. Sterile forceps were used to collect 1 to 2 feathers from the breast, dorsum, wings, and tail of each chick. All feathers from one bird were combined and placed in a 2-mL ZR BashingBead™ Lysis Tube and stored on ice. Intestinal contents from the entire GI tract (from the duodenum to cecum) were aseptically collected by squeezing and pooling the digesta from the duodenum to cecum into a ZR BashingBead™ Lysis Tube and snap frozen in liquid nitrogen. A swab pre-soaked in PBS was used to obtain a mycobiome sample from the transportation box. On days 3 and 7, two additional wood shaving samples were obtained, and 20 chickens were euthanized on each sampling day, followed by collection and pooling of the intestinal contents from the duodenum to cecum.

### Sample processing

All environmental samples were stored on ice and immediately transferred to the lab for further processing. Each feed (5 g) or wood shaving sample (10 g) was mixed with 45 and 90 mL sterile PBS, respectively, and shaken thoroughly on an orbital shaker at 400 RPM for 20 min to dissociate the microbes from solid matrices. Solutions were then transferred to 50-mL conical tubes by filtering through 4 layers of autoclaved cheesecloth. Feed, wood shavings, water, and air dust samples were then centrifuged at 2800×*g* for 5 min at 4 °C. Supernatants were removed and pellets resuspended with 750 μL BashingBead Buffer (Zymo Research). Microbial suspensions were transferred to ZR BashingBead™ Lysis Tubes and stored at – 80 °C. Tips from cotton swabs used to collected cage and transportation box samples were placed directly in ZR BashingBead™ Lysis Tubes and stored at – 80 °C.

### DNA isolation and sequencing

Microbial DNA was isolated from the digesta and environmental samples using the ZR *Quick-*DNA Fecal/Soil Microbe Kit (Zymo Research) according to the manufacturer’s protocol. DNA quality and quantity were determined using Nanodrop, and the absence of degradation was confirmed using agarose gel electrophoresis. High-quality DNA samples with the A_260_/A_280_ ratio ≥ 1.80 showing no or minimum degradation on agarose gels were shipped on dry ice to Novogene (Beijing, China) for PE250 sequencing of the ITS2 region of fungal rRNA genes using ITS3 (GCATCGATGAAGAACGCAGC) and ITS4 (TCCTCCGCTTATTGATATGC) primers on an Illumina HiSeq platform. PCR amplification and library preparation were performed by Novogene using NEBNext® Ultra™ DNA Library Prep Kit (New England Biolabs, Ipswich, MA). A DNA extraction control was also sequenced as a negative control.

### Data processing

Raw fungal sequences were imported into QIIME 2 [28] and processed using Deblur [29]. The ‘deblur denoise-other’ option with truncated sequences of 340 nucleotides was utilized through positive alignment-based filtering against the UNITE reference database [30]. Denoised sequences, often referred to as amplicon sequence variants (ASVs), were further classified using the WARCUP v2 fungal ITS database and the Ribosomal Database Projects (RDP) Bayesian Classifier [31, 32]. Bootstrap confidence was set to 80% for every taxonomic level and ASVs with a classification of less than 80% were assigned the name of the last confidently assigned level followed by “_unidentified”. Species classifications of the 25 most abundant ASVs were further confirmed through BLASTN search of the nucleotide database of GenBank. ASVs appearing in less than 5% of samples were removed from downstream analysis. Data was normalized using cumulative sum scaling in the metagenomeSeq package of R to correct bias in sampling depth across samples [33]. Analysis and visualization of the mycobiota composition was conducted in R version 3.5.1 [34].

Shannon index, Observed ASVs, and Pielou’s Evenness index were calculated to measure overall α-diversity, richness, and evenness of the mycobiota, respectively, using the phyloseq package version 1.24.2 in R [35]. Plots were made using ggplot2 version 3.0.0 [36]. Results were plotted using box and whisker plots, in which the middle line denotes the median value and the lower and upper hinges represent the first and third quartiles, respectively. Whiskers extend from the hinge to the highest or lowest value no farther than 1.5× the inter-quartile range. Points outside of this range are considered outliers. The β-diversity for evaluating differences among the mycobiota communities was calculated using weighted and unweighted UniFrac indices in the phyloseq package of R [35]. Dissimilarities were plotted as PCoA plots using phyloseq and ggplot2 in R. In order to identify potential microbial sources during the first week of life, non-normalized, unfiltered data from environmental and pooled intestinal samples was analyzed using SourceTracker [37] in R.

### Quantification of absolute abundance of the fungal and bacterial populations

Total populations of the fungi and bacteria in the chicken intestinal tract in the biogeography and succession studies were estimated using Femto™ Fungal and Bacterial DNA Quantification Kits (Zymo Research), respectively, according to the manufacturer’s protocols. Standard curves for fungi and bacteria were generated using quantitative PCR analysis of serial 10-fold dilutions of genomic DNA from *S. cerevisiae* strain TMY18 and *E. coli* strain JM109, respectively. Universal primers targeting ITS1 and the V4 region of the 16S rRNA gene were used for fungal and bacterial quantifications, respectively. Standard curves for fungal and bacterial DNA were calculated in this study to be *y* = −4.016*x* + 18.521 (*R*^2^ = 0.997) and *y* = −3.752*x* + 21.096 (*R*^2^ = 0.995), respectively, where *y* is the threshold cycle and *x* is log_10_ (ng of fungal or bacterial DNA). Total fungal or bacterial populations were further estimated using the following formula as recommended by the manufacturer: Total genome copy number = DNA (g) / (g-to-bp constant × genome size), where the g-to-bp constant is 1.096 × 10^−21^ g/bp and the average genome size of fungi and bacteria is 40 Mb [38] and 3.87 Mb [39], respectively. Results were expressed as fungal or bacterial genome copy number/g digesta, and the ratio of total genome copies of fungi to that of bacteria was further calculated for individual animals.

### Statistical analysis

Statistical significance was measured using parametric or nonparametric methods, depending on the normality of data and equality of group variances as determined by Shapiro-Wilk test and Brown-Forsythe test, respectively. While total fungi, total bacteria, and the fungal/bacterial ratio were subjected to ANOVA and post hoc Tukey test, nonparametric statistics was used when the data were not normally distributed. Significant differences in α-diversity and relative abundance were determined using Kruskal–Wallis test, followed by pairwise comparisons using Mann–Whitney *U* test. Pairwise R and *P* values of unweighted and weighted UniFrac distances were determined using ANOSIM in mothur with 999 permutations [40]. The false discovery rate (FDR) for relative abundance was controlled at 0.05 using the Benjamini-Hochberg procedure, while *P* < 0.05 was considered statistically significant in all other tests.

## Results

### Biogeography of the chicken intestinal mycobiome

Microbial DNA was isolated from a total of 350 digesta samples of seven different GI locations (the crop, ventriculus, duodenum, jejunum, ileum, cecum, and colon) of day-42 broiler chickens (Table S1) and subjected to ITS2 sequencing. A total of 26,226,549 quality filtered sequences, with an average of 62,295 sequences per sample and a range from 5652 to 117,794 sequences, were obtained. Following denoising using deblur, a total of 3290 ASVs were identified. After removal of rare ASVs present in less than 5% of samples, 329 ASVs were retained and included in downstream analysis.

**Fig. 1:**
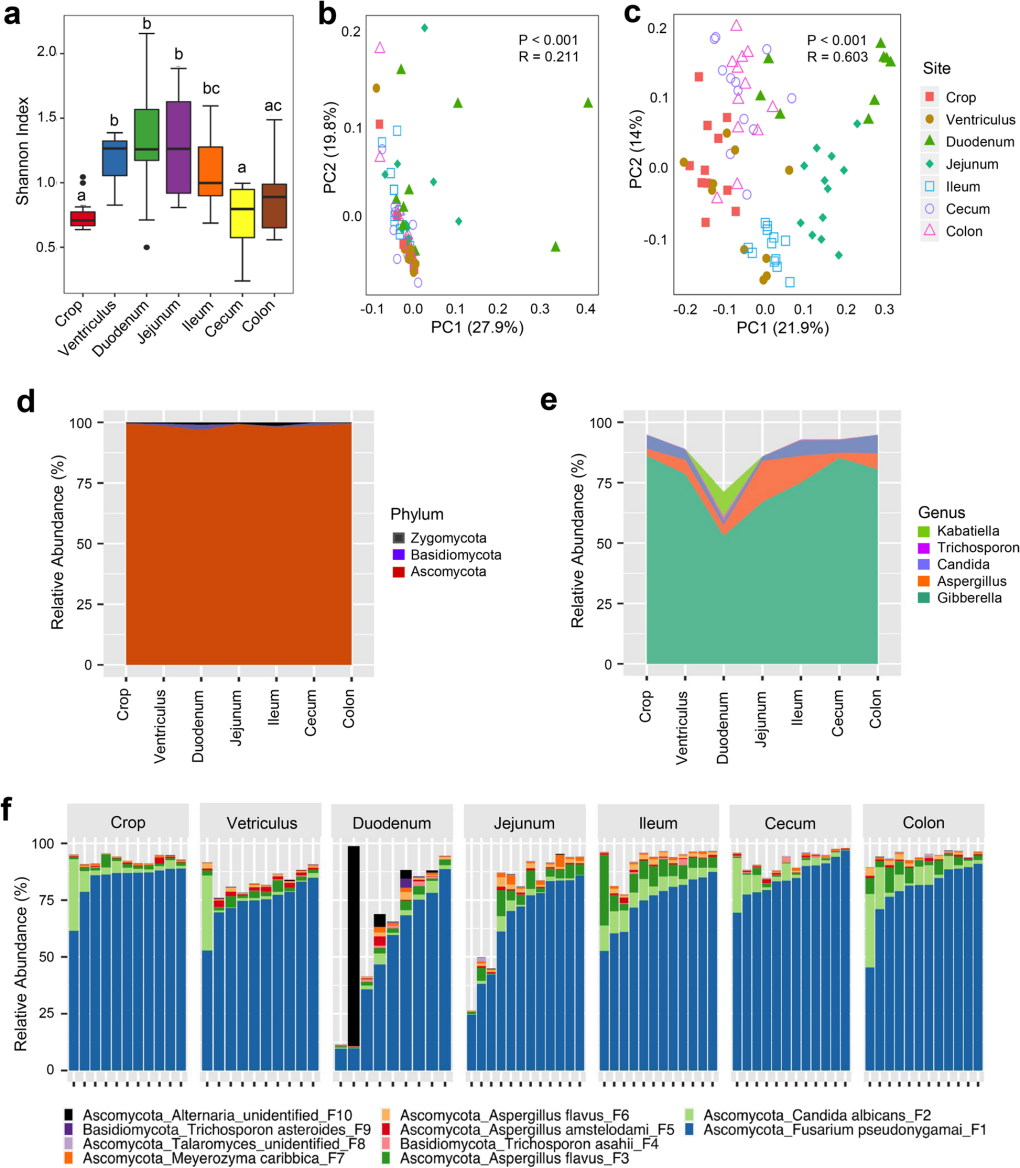
Diversity and biogeography of the intestinal mycobiome of day-42 chickens. The digesta was collected from different gastrointestinal segments from 12 broiler chickens on day 42 and subjected to DNA isolation and ITS2 sequencing. **a** Shannon Index showing α-diversity of the intestinal mycobiome. Significance was determined using Kruskal–Wallis test and post hoc Mann–Whitney *U* test. The bars not sharing a common superscript are considered significantly different (*P* < 0.05). PCoA of weighted (**b**) and unweighted UniFrac distances (**c**) was plotted to display β-diversity of the mycobiome in different intestinal segments. *R* and *P* values were calculated using ANOSIM with 999 permutations and indicated in each plot. Mean relative abundances (%) of the mycobiota in different segments of the gastrointestinal tract are displayed at the phylum (**d**) and genus (**e**) levels, while relative abundances (%) at the ASV level (**f**) were indicated by individual animals. Only the five most abundant genera and ten most abundant ASVs are shown

Evaluation of α-diversity using Shannon index revealed a significant variation (*P* < 0.05) along the GI tract (Fig. 1a). The lowest diversity was observed in the crop and increased in the ventriculus before reaching a maximum in the duodenum and jejunum. Diversity then decreased in the lower GI tract with the cecum and colon returning to levels similar to the crop. This trend was largely true with richness and evenness measurements as indicated by Observed ASVs and Pielou’s Evenness indices, respectively (Fig. S1). Statistical analysis using ANOSIM further revealed a significant difference (*P* < 0.001) in both weighted UniFrac (Fig. 1b) and unweighted UniFrac (Fig. 1c) indices of β-diversity. Pairwise comparisons of weighted and unweighted UniFrac distances were significantly different (*P <* 0.05) between all pairs of GI segments except between the cecum and colon (Table S2). The mycobiome in different GI segments were apparently more segregated in unweighted than weighted UniFrac (Fig. 1b, c), suggesting the presence of a number of rare bacteria unique to each segment.

Ascomycota was revealed to be the dominant phylum accounting for 97–99% of sequences in each GI location (Fig. 1d). A total of 81 genera were identified in the GI tract of day 42-old chickens. *Gibberella* was the most abundant genus accounting for greater than 50% of all sequences in each segment, followed by *Aspergillus* and *Candida* (Fig. 1e)*.* In fact, the five most abundant genera comprised nearly 95% of the sequences in all GI locations except for the duodenum, where they made up greater than 70% (Fig. 1e). At the ASV level, three ASVs were dominant and comprised more than 80% of the sequences in each location, with the exception of the duodenum and jejunum, where they accounted for 56% and 73%, respectively (Fig. 1f and Table S3). *Fusarium pseudonygamai* (F1), a member of *Gibberella*, was the most abundant, followed by *Candida albicans* (F2) and *Aspergillus flavus* (F3).

Statistical analysis on relative abundances of top 25 abundant ASVs revealed a significant difference among all GI segments (FDR < 0.05) except for *Trichosporon asteroids* (F9) and *Phialemonium curvatum*_(F24) (Table S3). In accordance with the α-diversity calculations, most significant differences were associated with increased diversity in the upper GI tract. Specifically, relative abundances of *F. pseudonygamai* (F1) and *C. albicans* (F2) decreased in the ventriculus and duodenum, relative to the crop, before increasing steadily from the jejunum to colon (Fig. 1f and Table S3). Notably, a decrease in dominant fungal taxa was associated with a concomitant increase in several less abundant ASVs in those locations. Relative to the crop, a bloom of *Alternaria* (F10), *Davidiella* (F14), *Aureobasidium pullulans* (F20), *Kabatiella* (F21), and *F. annulatum* (F25) was observed in the ventriculus or duodenum (Table S3), which is consistent with increased α-diversity in both locations.

**Fig. 2:**
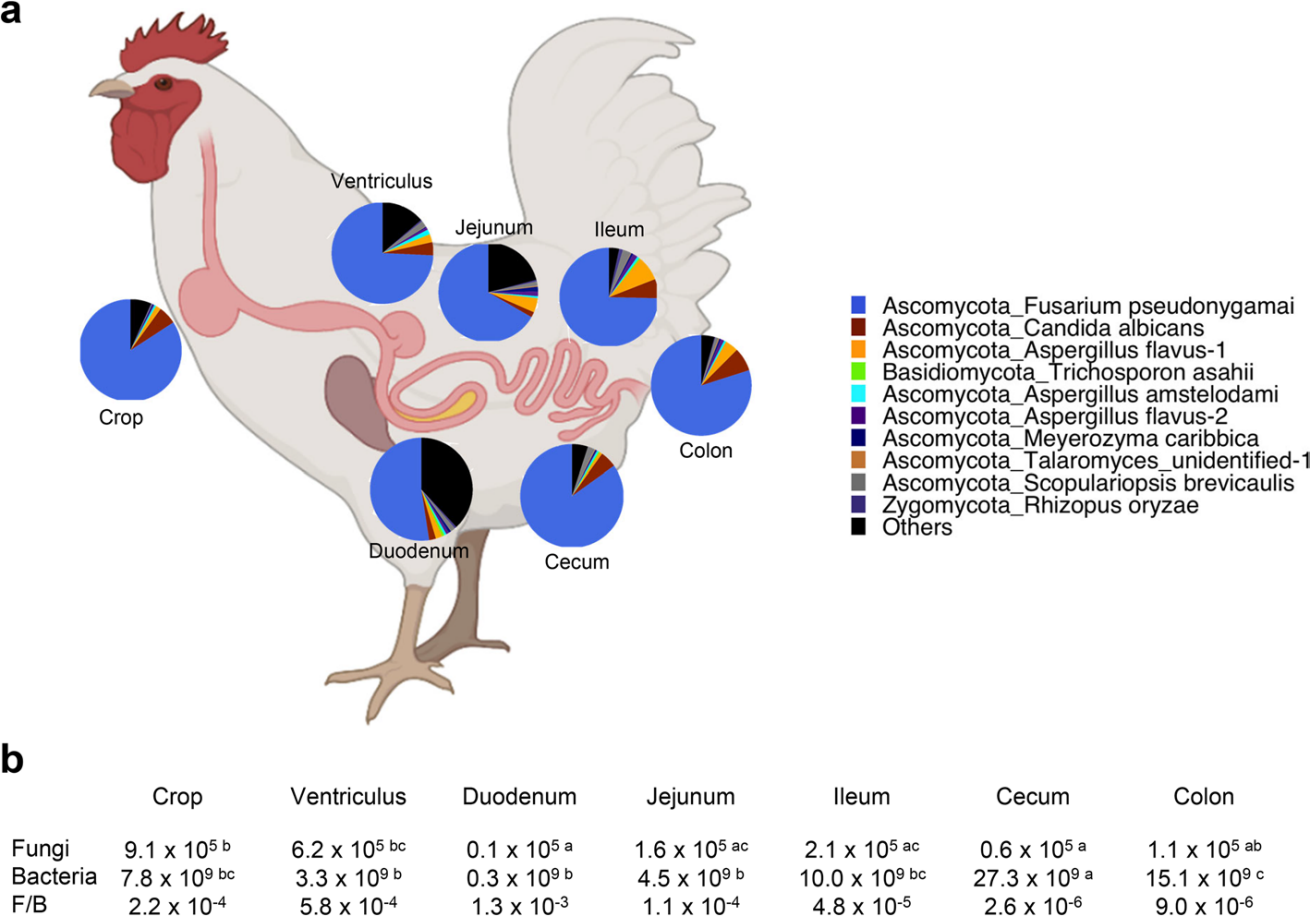
Biogeography of the core mycobiome and microbial burden along the gastrointestinal tract of day-42 chickens. **a** Relative abundances (%) of the ten most abundant core fungal ASVs. **b** Absolute abundances (genome copy number/g digesta) of the fungal and bacterial populations as well as the ratios of the fungal to bacterial genome copy number. Statistics was performed with ANOVA and post hoc Tukey test. The values in a row not sharing a common superscript are considered significantly different (*P* < 0.05)

Because of large variations in the composition of the mycobiome and presence of a number of rare unique ASVs among individual animals, we sought to identify a core mycobiome and compare their differences among different GI segments. In this study, the core mycobiome was defined as fungi ASVs present in at least 80% of samples at a given time and a GI location. A total of 26 ASVs were identified as core fungal taxa and accounted for more than 96% of the sequences in every GI location except for the duodenum and jejunum, where they represented approximately 85% of the sequences (data not shown). The core mycobiome was dominated by *F. pseudonygamai*, *C. albicans*, and *A. flavus* throughout the GI tract (Fig. 2a)*.* The remaining ASVs belonged primarily to the phyla Ascomycota (Fig. 2a). It is worth noting that top 25 abundant fungal ASVs were found to be mostly preserved in the core mycobiome.

### Succession of the chicken intestinal mycobiome

**Fig. 3:**
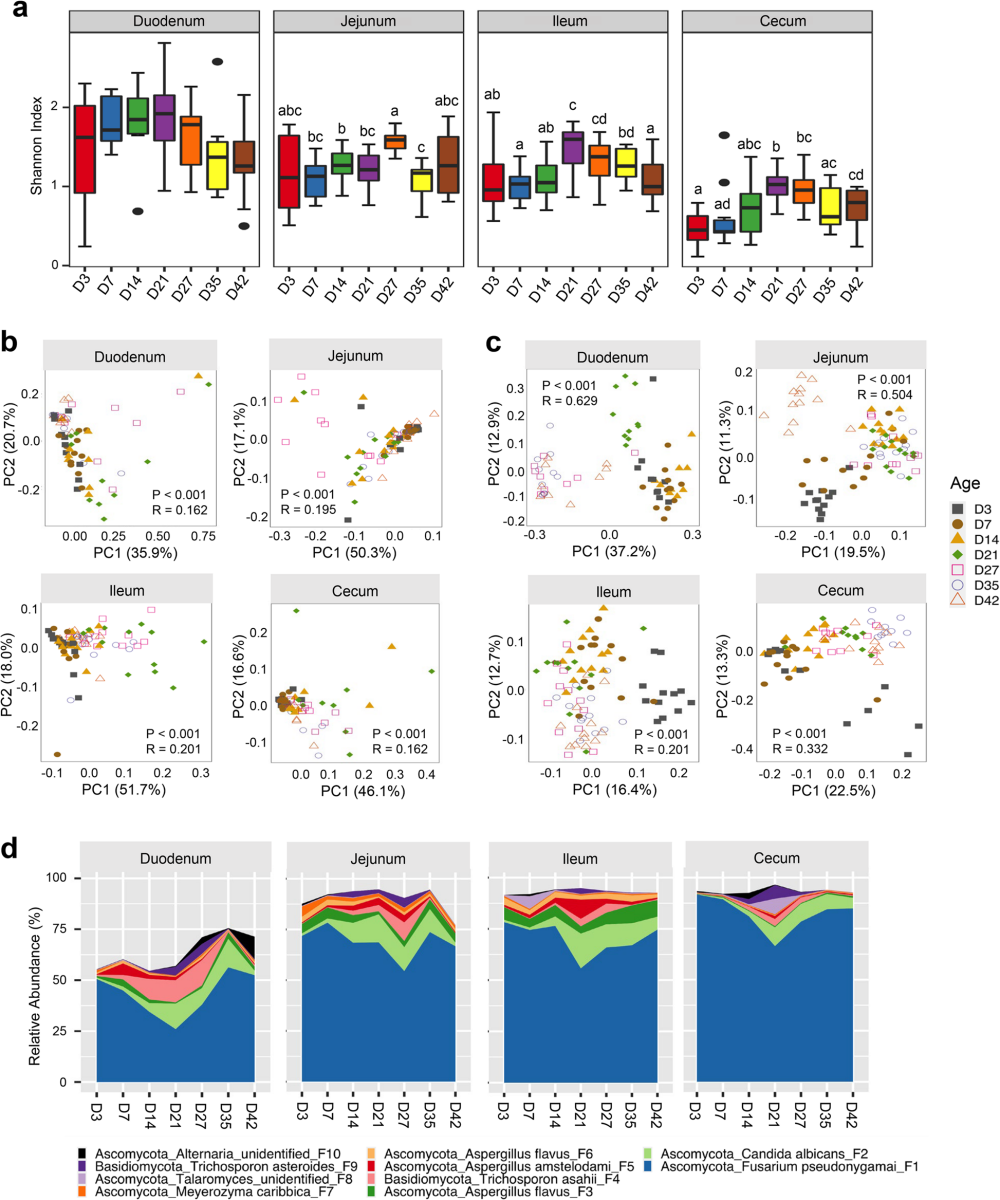
Successional changes in diversity and relative abundance of the chicken intestinal mycobiota throughout a 42-day production cycle. The digesta was collected from four different intestinal segments at seven different time points with 12 broilers per time point. **a** Shannon Index showing α-diversity of the intestinal mycobiome. Significance was determined using Kruskal–Wallis test and post hoc Mann–Whitney *U* test. The bars not sharing a common superscript are considered significantly different (*P* < 0.05). PCoA of weighted (**b**) and unweighted UniFrac distances (**c**) was plotted to display β-diversity of the intestinal mycobiome at different ages. *R* and *P* values were calculated using ANOSIM with 999 permutations and indicated in each plot. **d** Mean relative abundances (%) of the ten most abundant fungal ASVs at different ages in four different intestinal segments

To evaluate the dynamics of the intestinal mycobiome throughout an entire production cycle of broiler chickens, the digesta samples were obtained from the duodenum, jejunum, ileum, and cecum on days 3, 7, 14, 21, 27, 35, and 42 and subjected to DNA isolation and ITS2 sequencing. Shannon Index revealed significant changes in overall α-diversity in the jejunum, ileum, and cecum as chickens aged (Fig. 3a). In the ileum and cecum, diversity appeared to be relatively consistent on days 3, 7, and 14 before increasing significantly on day 21. A steady decrease was observed from day 27 to 42 (Fig. 3a). This pattern was less clear in the jejunum; however, a sharp increase in diversity was noted on day 27 followed by a decrease on day 35. Diversity in the jejunum recovered slightly on day 42. A similar trend was also observed for the duodenum, albeit with no significant difference in Shannon Index in the first 42 days (Fig. 3a), which appeared to be driven by opposite changes in species richness and evenness.

In the duodenum, richness of the mycobiota measured by Observed ASVs (Fig. S2a) remained relatively low from day 3 to day 21 before increasing significantly on days 27 to 42, whereas Pielou’s Evenness Index was sharply decreased beginning on day 27 and becoming even more pronounced on days 35 and 42 (Fig. S2b). In the jejunum, no significant differences in species richness were observed, while evenness remained stable except for a significant increase on day 27 (*P* < 0.05). Richness varied only slightly in the ileum and cecum throughout a 42-day production cycle of broiler chickens with significant differences found only in the cecum (Fig. S2a). Pielou’s Evenness Index more closely resembled that of Shannon Index for both the ileum and cecum indicating that diversity in these samples is driven by changes in species evenness rather than richness (Fig. S2b).

The β-diversity analysis revealed significant (*P* < 0.05) changes in fungal composition as chickens aged. However, *R* values for weighted UniFrac (Fig. 3b) were lower than those for unweighted UniFrac (Fig. 3c) for each GI location, suggesting an appearance of rare species in each location during the development of chickens. These results were confirmed by pairwise ANOSIM (Table S4), which revealed most age-wise comparisons to be significantly different (*P* < 0.05).

Compositionally, Ascomycota and Basidiomycota were predominant in every GI segment regardless of age (Fig. S3a). At the genus level, *Gibberella* remained dominant in every segment regardless of age, followed by *Aspergillus* and *Candida*, although relative abundance of each varied widely between locations and across ages (Fig. S3b)*.* A slight bloom of Basidiomycota was observed on days 14 to 27, which appeared to be driven by an increase in members of the genus *Trichosporon* between days 14 and 27, with relative abundance increasing from less than 0.6% on day 3 to as much as 12.4% in the duodenum on day 27 (Table S5).

**Fig. 4:**
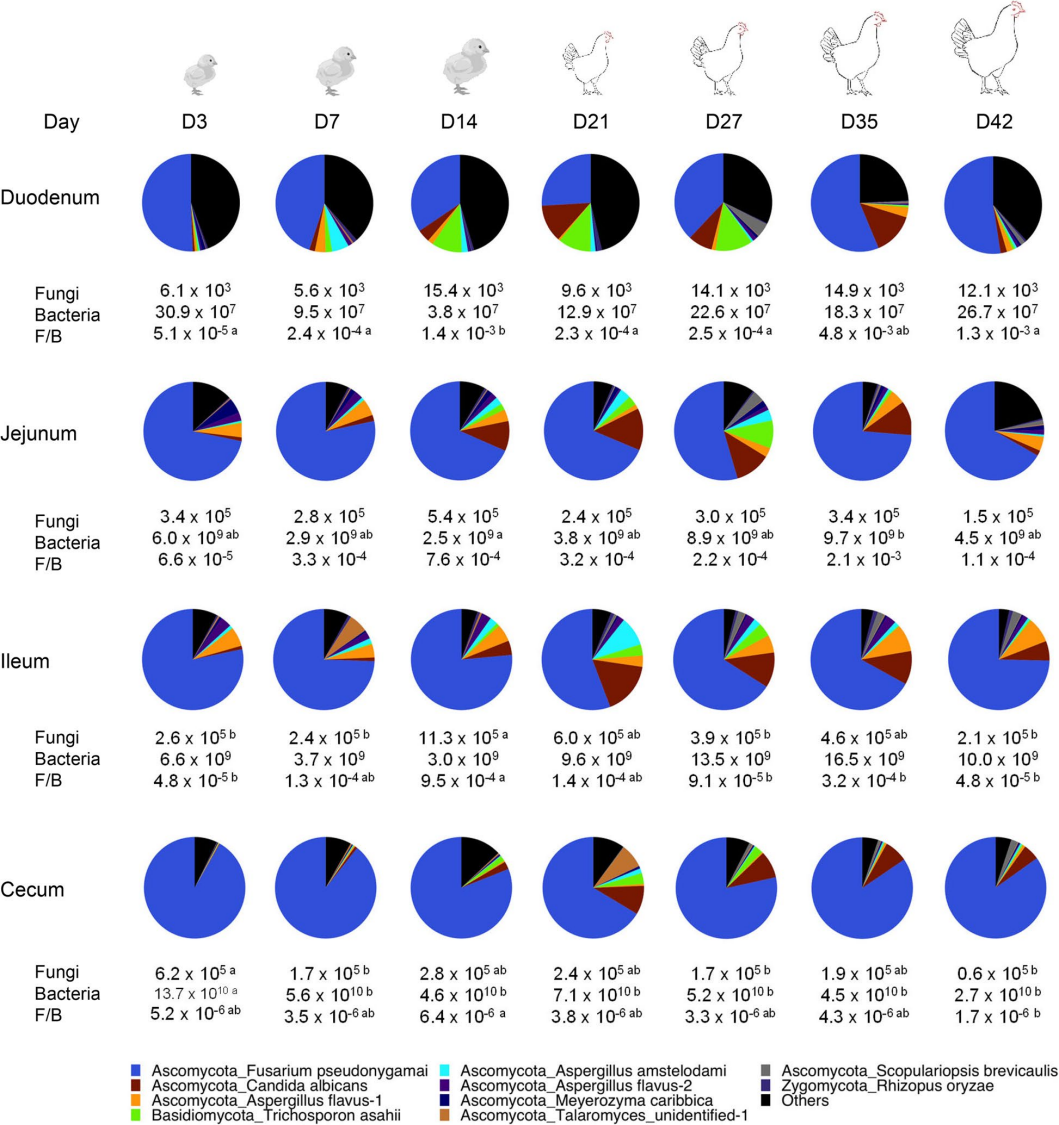
Succession and microbial burden of the chicken core intestinal mycobiome. Mean relative abundances (%) of the ten most abundant core fungal ASVs are indicated in pie charts, while absolute abundances (genome copy number/g digesta) of the fungal and bacterial populations as well as the ratios of the fungal to bacterial genome copy number are also shown below each segment on each sampling day. Statistics was performed with ANOVA and post hoc Tukey test. The values in a row not sharing a common superscript are considered significantly different (*P* < 0.05)

Investigation of fungal ASVs revealed obvious changes in fungal composition in each GI location as chickens aged (Fig. 3d). Consistent with the biogeography results, *F. pseudonygamai* (F1) was the dominant ASV throughout an entire production cycle in each GI segment. However, its abundance largely decreased in every location from day 3 to day 21 before recovering on days 35 and 42 (Fig. 3d). On the other hand, *C. albicans* (F2), two *Trichosporon* members (*T. asahii* F4 and *T. asteroids* F9) generally increased from day 3 to day 42. Statistical analysis on top 25 abundant ASVs revealed significant age-dependent differences (FDR < 0.05) in relative abundance of most ASVs in the duodenum, jejunum, ileum, and cecum (Table S5), suggestive of a dynamic development of the chicken intestinal mycobiome. Among the 10 most abundant ASVs present in the core microbiome of the chicken GI tract in a 42-day production cycle, *F. pseudonygamai* apparently predominated the chicken intestinal mycobiota, while *C. albicans*, two *Aspergillus* species (*A. favus* and *A. amstelodami)*, and two *Trichosporon* species (*T. asahii* and *T. asteroids*) also persisted in each GI location from day 3 to day 42 (Fig. 4).

### Fungal and bacterial population changes in the chicken GI tract

**Fig. 5:**
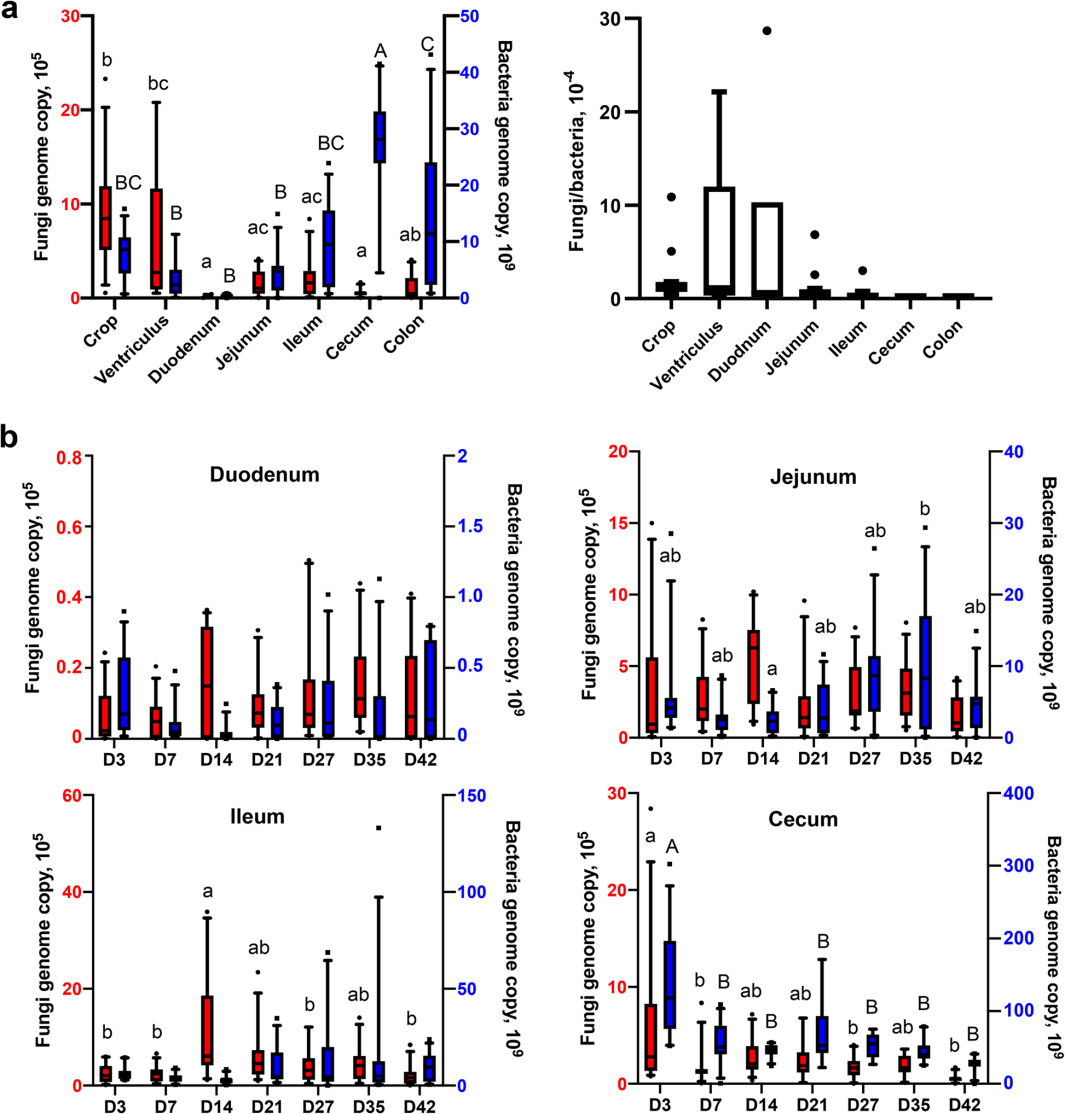
Absolute abundance of the chicken intestinal mycobiota and microbiota. Absolute abundances (genome copy number/g digesta) of the fungal and bacterial populations are shown along the gastrointestinal tract of day-42 broilers (**a**) and in four intestinal segments on different sampling days (**b**) with 12 samples per bar. Statistics was performed with ANOVA and post hoc Tukey test. The fungal (red ) and bacterial (blue) bars not sharing a common superscript are considered significantly different (*P* < 0.05)

To evaluate the changes in total populations of fungi and bacteria in different GI segments of day-42 broilers, qPCR was performed using universal primers separately for fungi and bacteria. Microbial burden varied considerably along the GI tract of broilers with opposite trends observed for fungi versus bacteria (Fig. 5a). The fungal population was shown to be highest in the crop and ventriculus at 9.1 × 10^5^ and 6.2 × 10^5^/g digesta, respectively, before decreasing significantly (*P* < 0.05) in the duodenum (0.1 × 10^5^/g) and remaining low throughout the rest of the intestinal tract (Figs. 2b and 5a). Bacterial population decreased from 7.8 × 10^9^/g digesta in the crop to 0.3 × 10^9^/g in the duodenum before increasing gradually to a maximum of 2.7 × 10^10^/g in the cecum. Relative to the bacterial population, fungi only accounted for no more than 0.06% in any location (Figs. 2b and 5a).

Dynamic changes in the fungal and bacterial populations at four GI locations (the duodenum, jejunum, ileum, and cecum) were further investigated throughout an entire production cycle using qPCR (Fig. 5b). The fungal population appeared to decrease from days 3 to 7 in each segment before reaching a maximum on day 14 and then decreasing again on day 21. The fungal population largely recovered slightly on days 27 and 35 before decreasing on day 42 in all four segments. Chickens appeared to have the lowest fungal load on day 42. On the other hand, the bacterial population generally increased in the duodenum, jejunum, and ileum as chickens aged, while the cecum followed an opposite trend (Figs. 4 and 5b).

### Origin of the chicken intestinal mycobiota

In order to identify the major factors contributing to an initial establishment of the chicken intestinal mycobiome, various environmental samples as well as the intestinal contents from the first week of chickens were collected. Swabs of the transportation box and feathers of day-of-hatch chicks were used to determine the impact of the hatchery environment, while wire mesh pen swabs, feed, airborne dust samples, and drinking water were collected from the poultry house prior to chickens’ arrival. Wood shavings and the entire intestinal contents were also collected on days 0, 3, and 7 to evaluate their respective influence shaping the early intestinal mycobiome.

**Fig. 6:**
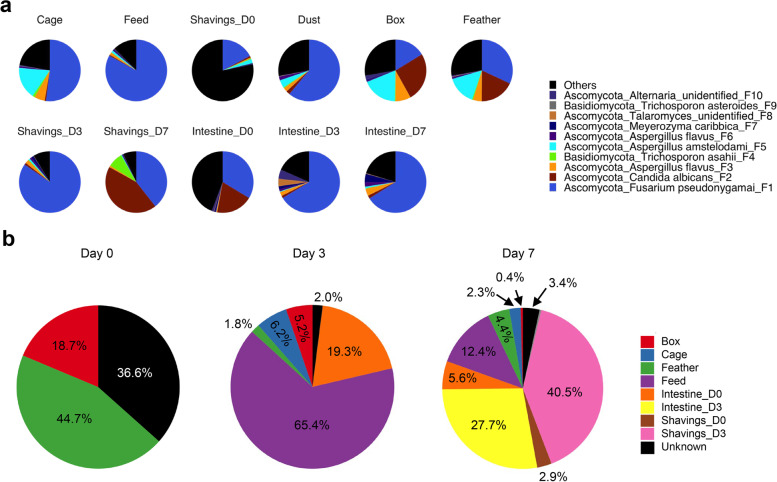
Sources of the chicken intestinal mycobiota. **a** Mean relative abundances (%) of the ten most abundant fungal ASVs in different environmental and pooled intestinal samples. **b** Mean relative contributions (%) of different environmental and pooled intestinal sample types to the initial establishment of the chicken intestinal mycobiota estimated using SourceTracker [37]

Among the 10 most abundant ASVs of environmental and intestinal samples, *F. pseudonygamai* (F1) was ubiquitously present in a majority of the samples. *F. pseudonygamai* accounted for 82.9%, 51.9%, and 17.3% of the fungal population in the feed, cage, and wood shavings, respectively (Fig. 6a). Transportation box and feather exhibited lower levels of *F. pseudonygamai* (16.3% and 32%), but higher relative abundance of *C. albicans* (25.6% and 18.1%), *A. flavus* (8.1% and 4.6%), and *A. amstelodami* (18.6% and 15.3%). Interestingly, the GI tract of day-of-hatch chicks harbored a diverse mycobiota with *F. pseudonygamai* and *C. albicans* accounting for 33.7% and 19.2% of the sequences, respectively, while 21.5% of the sequences consisted of fungal species outside of top 10 abundant ASVs (Fig. 6a). During the first week of life, a decrease in intestinal mycobiome diversity was observed which was associated with an increase in the relative abundance of *F. pseudonygamai*. Additionally, fungal composition of the wood shavings changed drastically with bedding on day 3 dominated by *F. pseudonygamai* (84.7%), while day-7 shavings were comprised of almost equal portions of *F. pseudonygamai* (39.4%) and *C. albicans* (43.7%) (Fig. 6a)*.*

SourceTracker [37] was further utilized to identify the sources of individual fungal species during the first week of life. Since day-of-hatch chickens did not have access to the poultry housing environment prior to collection of the intestinal contents, only transportation box and feather samples were included as potential sources in the day-0 analysis. Among these, feather accounted for 44.7% of the initial mycobiota and transportation boxes contributed 18.7% (Fig. 6b). However, 36.6% of the mycobiota came from unknown sources, presumably from the eggshells and the hatchery environment similar to what was revealed on the origin of the chicken intestinal microbiota [41]. Feed and day-0 intestinal mycobiota were identified as the primary fungal sources of the day-3 mycobiota contributing 65.4% and 19.3%, respectively, while the fungi on the feather and box diminished quickly to 1.8% and 5.2%, respectively (Fig. 6a). The influence of feed and initial mycobiome was reduced to combined 18.0% on the day-7 intestinal mycobiota, while day-3 wood shavings (40.5%) and intestinal mycobiome (27.7%) became the major contributors (Fig. 6a).

### Variabilities of the chicken intestinal mycobiota among different studies

Relative to the intestinal microbiota, the mycobiota in the GI tract appears to vary greatly among different studies in humans, mice, and chickens [7–10, 20–25]. To address the variations in the mycobiota composition between our current and previously published studies [27], we directly compared the differences in the core mycobiomes that are present in no less than 80% samples at a given GI location of day-28 broilers in respective studies. PCoA analysis of both unweighted and weighted UniFrac revealed intestinal samples to cluster separately by trial (Fig. S4a and b). However, a closer examination of individual core fungal species revealed an overlap of a small subset of the species present in both studies, while more than a half number of fungal ASVs were unique to each (Fig. S4c). *F. pseudonygamai*, *S. brevicaulis T. asteroides*, *S. maydis*, *A. amstelodami*, *A. flavus*, and *D. hansenii* were consistently present in all four GI locations examined in both studies (Fig. S4c). It is noted that these were also the fungi that were frequently reported in several earlier studies [20–25], suggesting that these species may be particularly well adapted to survive in the GI tract of chickens. *C. albicans* was the second most abundant fungal species revealed in this study and commonly reported earlier [20–25]. However, it was present in slightly less than 80% of the samples in our earlier study, *C. albicans* was, therefore, excluded from the core microbiota shared between the two studies.

## Discussion

Extensive studies of the microbiome over the past decade has highlighted the importance of the intestinal microbiome in health and disease and led to the development of new therapies [1, 2]. However, very little is known about the smaller microbial populations such as the mycobiota residing within the GI tract. In this study, we have systematically addressed the biogeography, succession, and origin of the intestinal mycobiota using the chicken as an animal model and we have made a number of important findings. We revealed that, similar to the microbiota, the mycobiota also shows clear differences in both the composition and total population in different segments of the GI tract. However, opposite to the microbiota that is more diverse in the lower GI tract, the mycobiota in the upper GI tract (crop, ventriculus, duodenum, jejunum, and ileum) is consistently more diverse than that in the lower GI tract (cecum and colon) of chickens. Similarly, the mycobiota was found to be the most diverse in the stomach and decreased in the small intestine before increased slightly in the colon of pigs [18]. In contrast to the intestinal microbiota that becomes more populated along the GI tract, total fungal population is more abundant in the crop and ventriculus than other lower GI locations. In fact, the crop and ventriculus harbor approximately 10 times more fungi than the cecum, while the opposite is largely true with the bacteria in chickens.

Relative to the crop, relative abundances of two most dominant fungi, *F. pseudonygamai* (F1) and *C. albicans* (F2) are decreased by 12–63% in the ventriculus or duodenum of day-42 chickens, which is associated with a concomitant increase in several less abundant fungi such as *Alternaria* (F10) and *Davidiella* (F14) (Table S3). However, the changes in relative abundances of lowly abundant fungi are unlikely to be a spurious result caused by the compositional nature of the data, because a great majority of rare fungal taxa are not proportionally increased and drastic changes are observed with many of them. For example, *Alternaria* (F10) only accounts for 0.04% of the total fungal population in the crop, but blooms markedly to 10.96% in the duodenum (Table S3). Even with a 91-fold difference in the total fungal population between the crop and duodenum (Fig. 2b), absolute abundance of *Alternaria* (F10) in the duodenum is still increased by more than 8-fold, relative to the crop. Moreover, *Davidiella* (F14), *A. pullulans* (F20), and *Kabatiella* (F21) are virtually absent in the crop, but are increased to 6.15%, 3.77%, and 6.42% in the duodenum, respectively (Table S3), suggesting that these changes are not computationally spurious, but consistent with high diversity of the mycobiome in the duodenum, which may play an important physiological role.

The intestinal microbiota is known to undergo successional changes and tends to become more diversified and stabilized as the host ages [42–45]. Although we observed a significant difference in both α- and β-diversities of the mycobiota in the duodenum, jejunum, ileum, and cecum over time, an age-dependent succession, diversification, or maturation of the chicken intestinal mycobiota was not evident. While the intestinal mycobiota tends to become more diverse up to 21 days post-hatch, its diversity is gradually reduced beyond 21 days. In accordance with our study, α-diversity of the ileal mycobiota was recently found to increase in turkeys from day 3 to day 13 [26]. The human and porcine intestinal mycobiota showed no clear pattern of age-dependent maturation as well [19, 46]. In fact, an inverse relationship between the intestinal fungal α-diversity and age was observed in an human study showing that infants and children harbor more fungi than adults [46]. Total fungal population remains relatively stable in the duodenum, jejunum, or ileum of chickens up to market age, while the bacterial population tends to increase over the age. However, both the cecal mycobiota and microbiota have a tendency to decrease during the development. In pigs, a culture-based approach confirmed that the fungal load was below the level of detection until day 21 and then increased significantly on days 28 and 35 [19]. It is noteworthy that the successional alterations of the intestinal mycobiota in this study is also likely influenced by dietary changes, in addition to host physiology.

As for the origin of the intestinal mycobiota, we previously suggested that it may originate from the feed and the housing environment based on possible sources of prevalent fungal species [27]. In this study, we directly assessed the origin of the chicken intestinal mycobiota by analyzing the feed and a variety of environmental samples. It is conceivable that the fungi on the feature and transportation box are major contributors of the intestinal mycobiota of newly hatched chicks. Although we could not directly access and analyze the hatchery mycobiota, it is apparent that the mycobiota on the feature and transportation box came from the hatchery environment such as egg incubators, eggshells, storage boxes, and human handlers, which collectively provided the initial fungal inoculum to chicks.

However, unlike the intestinal microbiota on which the bacteria on eggshells have a lasting influence [41], the initial mycobiota from the hatchery environment is quickly replaced within 3 days by those from the housing environment, particularly the feed, suggesting the importance of the diet in shaping the intestinal mycobiota. This is also consistent with the fact that all of those dominant intestinal fungi such as *Gibberella*, *Aspergillus*, and *Trichosporon* are commonly associated with the soil and plants [47–50]. For example, *F. pseudonygamai*, a *Gibberella* member and the predominant intestinal fungal species identified in this study, are frequently found in staple cereal crops such as corn and sorghum. Therefore, the mycobiota in the housing environment and the dietary ingredients in particular plays a critical role in influencing the structure and function of the chicken intestinal mycobiota. Given that many of these fungi such as *F. pseudonygamai* are plant pathogens and also capable of producing mycotoxins including fumonisins and moniliformin that are known to be detrimental to both humans and livestock animals [51], therefore, managing and minimizing the colonization of these pathogenic fungi will positively impact the health and productivity of the animals. On the other hand, even with *F. pseudonygamai* colonizing in the GI tract, the broilers are apparently healthy with no obvious intestinal abnormalities (data not shown). The role of low levels of seemingly pathogenic fungi on animal physiology and metabolism warrants further investigation.

Similar to earlier observations in other animal species [7–10], the chicken mycobiota shows great variations among different studies [20–25]. Culture-dependent studies typically report fewer than 20 fungal species, with *Candida* being frequently described as the most abundant genus in the GI tract of chickens and turkeys, although dominant *Candida* species varies by study [21–24]. *Trichosporon*, *Geotrichum*, *Rhodotorula*, and *Saccharomyces* have also been frequently isolated in chickens [21–23]. However, one other culture-dependent study reported the identification of 88 fungal species from over 3000 cecal samples of broiler and layer chickens, with *Aspergillus*, *Penicillium*, *Verticillium*, and *Sporidiobolus* being the four most abundant genera [25]. Culture-independent 454 pyrosequencing only revealed two *Cladosporium* species in the cecum of broilers fed unmedicated diets [20], presumably due to a lack of sequencing depth. More recently, next-generation sequencing identified *Sarocladium kiliense* to be abundant in the ileum of turkeys along with members of *Candida*, *Dothidea*, and *Cladosporium* [26].

Our earlier investigation of the chicken intestinal mycobiome based on Illumina HiSeq sequencing of the ITS2 region revealed the presence of 468 unique fungal ASVs with *Microascus* (*Scopulariopsis brevicaulis)* being predominant, followed by *Trichosporon asahii*, and *Aspergillus* [27]. However, in this study, *Gibberella (F. pseudonygamai*), *Candida* (*C. albicans*), two *Aspergillus* species (*A. flavus* and *A. amstelodami*), and *T. asahii* was found to be the five most abundant fungi*.* While *S. brevicaulis* was identified in this study, its relative abundance was much lower, ranging from 0.2 to 2.8% on day 42 (data not shown). This contrasts with a high abundance of *S. brevicaulis* observed in our earlier study [27], suggesting that, as a minor community, the intestinal mycobiota is highly influenced by the housing environment, particularly the fungi present in feed ingredients. Any fluctuations in the fungal community that chickens come into contact with are likely to alter the structure and function of the intestinal mycobiota. This also helps explain great variations existing among different studies when the housing environment and sources of feed ingredients are different.

## Conclusions

In summary, we comprehensively investigated the biogeography and succession of the intestinal mycobiota throughout an entire production cycle of broiler chickens. The chicken mycobiota is more diverse in the upper GI tract than the lower GI tract, with no apparent pattern of succession or maturation up to 42 days of age. The total fungal population represents less than 0.06% of the bacterial population in the GI tract. The chicken intestinal mycobiota is less diverse than the microbiota and dominated by only a few fungal species. Initial intestinal fungal inoculum is originated from the hatchery environment, but replaced quickly by the microbiota in the housing environment, particularly the feed. A better understanding of the biogeography, succession, and origin of the intestinal mycobiota will facilitate the development of novel approaches to managing and manipulating the intestinal fungal community to achieve better animal health and productivity.

## Supplementary Information

**Additional file 1: Fig. S1.** Alpha-diversity of the intestinal mycobiome of day-42 chickens. **Fig. S2.** Successional changes in ⍺-diversity of the chicken intestinal mycobiome throughout a 42-day production cycle. **Fig. S3.** Successional changes in relative abundance (%) of the chicken intestinal mycobiome throughout a 42-day production cycle. **Fig. S4.** Differences in the chicken core intestinal mycobiota between two studies. **Supplementary Table S1.** Types and quantities of the samples sequenced in this study. **Supplementary Table S2.** Pairwise ANOSIM of β-diversity of the intestinal mycobiota of day-42 chickens. **Supplementary Table S3.** Relative abundance (%) of the mycobiota in the gastrointestinal tract of day-42 chickens. **Supplementary Table S4.** Pairwise ANOSIM of β-diversity of the chicken intestinal mycobiota during development. **Supplementary Table S5.** Temporal shifts in the chicken mycobiota composition (%).

## Data Availability

The raw sequencing reads of this study have been deposited in NCBI under the accession number PRJNA512838.
