# Supplementary material for: Biogeography, succession, and origin of the chicken intestinal mycobiome
Source: Microbiome. 2022 Apr 1;10:55. doi: 10.1186/s40168-022-01252-9 (PMC8976367; doi:10.1186/s40168-022-01252-9)
Supplement: Supplementary file 2 — Additional file 1: Fig. S1. Alpha-diversity of the intestinal mycobiome of day-42 chickens. Fig. S2. Successional changes in ⍺-diversity of the chicken intestinal mycobiome throughout a 42-day production cycle. Fig. S3. Successional changes in relative abundance (%) of the chicken intestinal mycobiome throughout a 42-day production cycle. Fig. S4. Differences in the chicken core intestinal mycobiota between two studies. Supplementary Table S1. Types and quantities of the samples sequenced in this study. Supplementary Table S2. Pairwise ANOSIM of β-diversity of the intestinal mycobiota of day-42 chickens. Supplementary Table S3. Relative abundance (%) of the mycobiota in the gastrointestinal tract of day-42 chickens. Supplementary Table S4. Pairwise ANOSIM of β-diversity of the chicken intestinal mycobiota during development. Supplementary Table S5. Temporal shifts in the chicken mycobiota composition (%). [file 40168_2022_1252_MOESM2_ESM.docx]

**
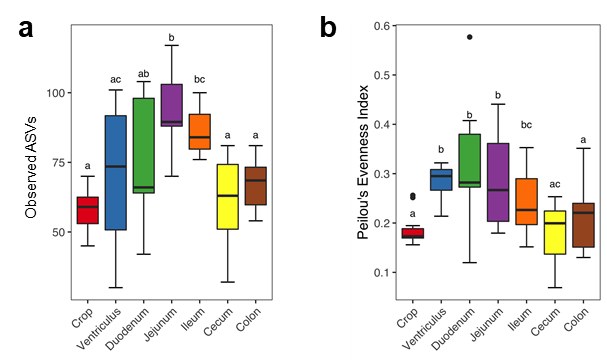
**

**Fig. S1. Alpha-diversity of the intestinal mycobiome of day-42 chickens.** (**a**) Observed ASVs indicating richness. (**b**) Pielou’s Evenness index showing evenness. Statistical differences were determined using Kruskal-Wallis test and post-hoc Mann-Whitney U test. The bars not sharing a common superscript are considered significantly different (P < 0.05).


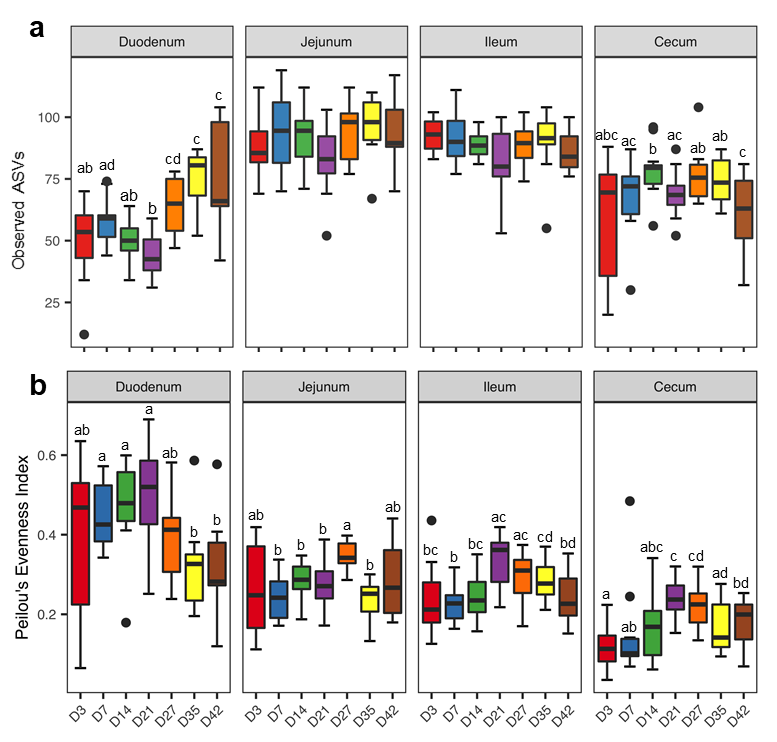


**Fig. S2. Successional changes in ⍺-diversity of the chicken intestinal mycobiome** **throughout a 42-day production cycle.** (**a**) Observed ASVs indicating richness. (**b**) Pielou’s Evenness index showing evenness. Statistical differences were determined using Kruskal-Wallis test and *post-hoc* Mann-Whitney U test. The bars not sharing a common superscript are considered significantly different (P < 0.05).


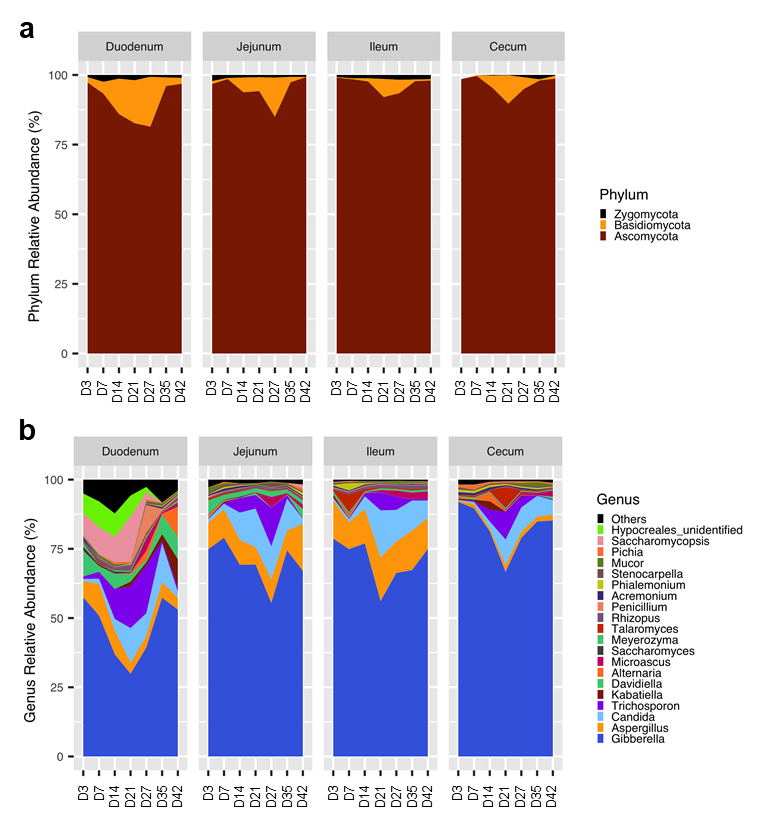


**Fig. S3. Successional changes in relative abundance (%) of the chicken intestinal mycobiome throughout a 42-day production cycle.** Mean relative abundances (%) of top three abundant fungal phyla (**a**) and top 20 abundant genera (**b**) at different ages in four different intestinal segments are shown.

**
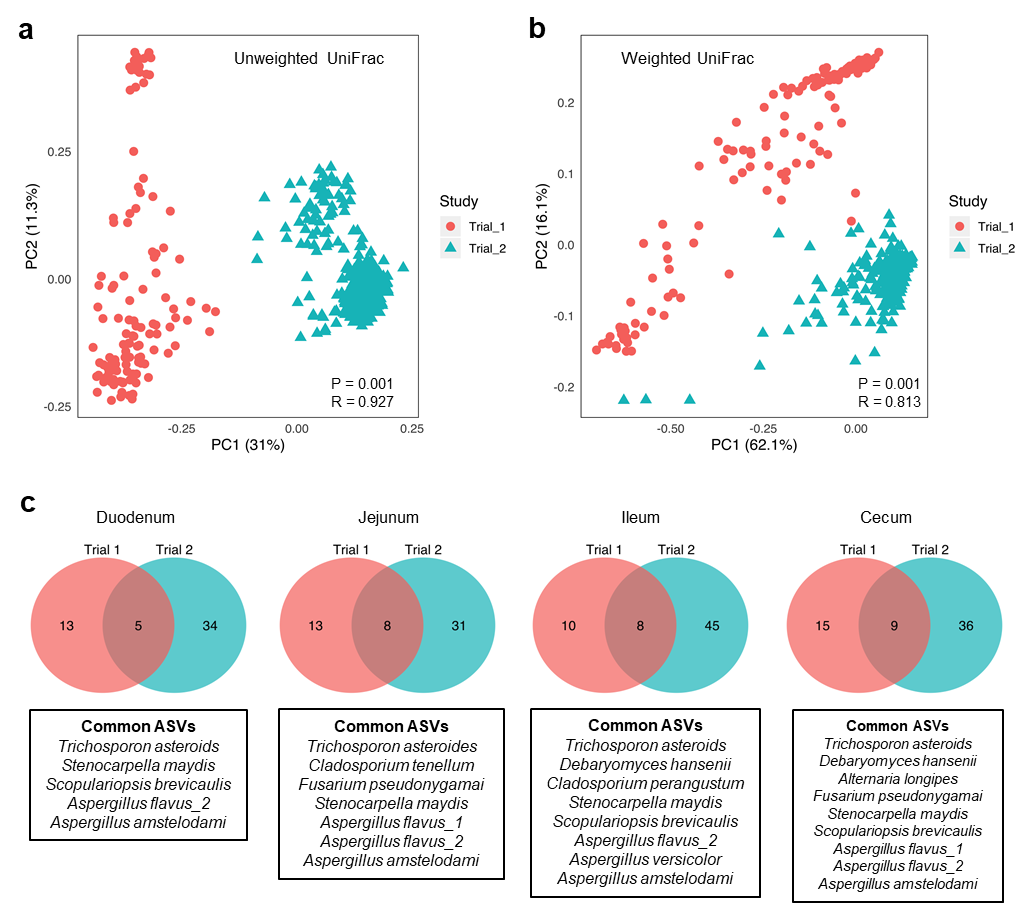
**

**Fig. S4. Differences in the chicken core intestinal mycobiota between two studies.** PCoA of unweighted (**a**) and weighted UniFrac distances (**b**) was plotted to indicate β-diversity of the intestinal mycobiome of day-28 broilers between an earlier study of ours [27] (Trial 1) and this current study (Trial 2). Note a clear segregation of the samples between the two studies. (**c**) Venn diagram showing the overlapping of the core fungal ASVs in each of four intestinal segments between the two studies. Common fungal ASVs are indicated below each intestinal segment.

**Supplementary Table S1. Types and quantities of the samples sequenced in this study**

| Sample Type | Chicken Age | Sample Quantity |  | Sample Type | Chicken Age | Sample Quantity |
| --- | --- | --- | --- | --- | --- | --- |
| For the biogeography and succession studies (n = 350) ^1^: | | | | | | |
| Duodenal digesta | D3 | 10 |  | Ileal digesta | D3 | 12 |
|  | D7 | 12 |  |  | D7 | 12 |
|  | D14 | 9 |  |  | D14 | 12 |
|  | D21 | 11 |  |  | D21 | 12 |
|  | D27 | 9 |  |  | D27 | 12 |
|  | D35 | 10 |  |  | D35 | 12 |
|  | D42 | 10 |  |  | D42 | 12 |
| Jejunal digesta | D3 | 12 |  | Cecal digesta | D3 | 12 |
|  | D7 | 12 |  |  | D7 | 12 |
|  | D14 | 12 |  |  | D14 | 12 |
|  | D21 | 12 |  |  | D21 | 10 |
|  | D27 | 11 |  |  | D27 | 10 |
|  | D35 | 12 |  |  | D35 | 10 |
|  | D42 | 12 |  |  | D42 | 12 |
| Crop digesta | D42 | 11 |  |  |  |  |
| Colonic digesta | D42 | 12 |  |  |  |  |
| Ventriculus digesta | D42 | 11 |  |  |  |  |
| For the mycobiome origin study (n = 78): | | | | | | |
| Pooling of intestinal digesta | D0 | 20 |  | Air dust^2^ | D0 | 1 |
|  | D3 | 20 |  | Tap water^2^ | D0 | 1 |
|  | D7 | 19 |  | Wood shavings | D0 | 2 |
| Feather | D0 | 5 |  | Wood shavings | D3 | 2 |
| Feed | D0 | 2 |  | Wood shavings | D7 | 2 |
| Cage | D0 | 2 |  | DNA extraction control^2^ |  | 1 |
| Transportation box | D0 | 1 |  |  |  |  |
| Total number of samples sequenced | | | | | | 428 |

^1^ A total of 12 digesta samples were initially collected from each of 12 animals, one per cage, for each GI location at each sampling time; however, a small number of DNA samples were of inadequate quality and thus excluded for sequencing.

^2^ Air dust and tap water samples from animal room were extracted for DNA, but failed for sequencing, together with the DNA extraction control sample.

**Supplementary Table S2: Pairwise ANOSIM of β-diversity of the intestinal mycobiota of day-42 chickens**

|  | Crop | Ventriculus | Duodenum | Jejunum | Ileum | Cecum | Colon |
| --- | --- | --- | --- | --- | --- | --- | --- |
| Crop |  | *< 0.001 (0.413)* | *< 0.001 (0.783)* | *< 0.001 (0.913)* | *< 0.001 (0.742)* | *< 0.001 (0.502)* | *< 0.001 (0.350)* |
| Ventriculus | < 0.001 (0.303) |  | *< 0.001 (0.692)* | *< 0.001 (0.704)* | *< 0.001 (0.683)* | *0.001 (0.662)* | *< 0.001 (0.529)* |
| Duodenum | < 0.001 (0.354) | < 0.001 (0.279) |  | *< 0.001 (0.626)* | *< 0.001 (0.815)* | *< 0.001 (0.688)* | *< 0.001 (0.633)* |
| Jejunum | < 0.001 (0.249) | 0.010 (0.163) | 0.029 (0.159) |  | *< 0.001 (0.676)* | *< 0.001 (0.821)* | *< 0.001 (0.739)* |
| Ileum | < 0.001 (0.408) | < 0.001 (0.374) | 0.001 (0.322) | 0.017 (0.094) |  | *< 0.001 (0.736)* | *< 0.001 (0.621)* |
| Cecum | 0.017 (0.119) | 0.002 (0.370) | < 0.001 (0.354) | 0.001 (0.189) | 0.001 (0.315) |  | *0.271 (0.027)* |
| Colon | 0.005 (0.154) | < 0.001 (0.300) | < 0.001 (0.311) | 0.021 (0.078) | 0.007 (0.140) | 0.083 (0.060) |  |

**Note:** P and R values of pairwise comparisons based on weighted and unweighted UniFrac distances were determined by ANOSIM with 999 permutations. R value for each comparison is shown in parenthesis. Those values for unweighted UniFrac are italicized.

**Supplementary Table S3: Relative abundance (%) of the mycobiota in the gastrointestinal tract of day-42 chickens**

| ASV | Crop | Ventriculus | Duodenum | Jejunum | Ileum | Cecum | Colon | P-Value | FDR |
| --- | --- | --- | --- | --- | --- | --- | --- | --- | --- |
| Ascomycota *Fusarium pseudonygamai*_F1 | 84.19^a^ | 74.27^bc^ | 52.44^b^ | 66.70^bd^ | 74.66^bd^ | 84.88^ac^ | 79.93^ad^ | <0.001 | 0.001 |
| Ascomycota *Candida albicans­_*F2 | 5.78^a^ | 4.19^b^ | 2.14^abd^ | 1.74^b^ | 6.29^c^ | 5.21^acd^ | 7.59^acd^ | 0.001 | 0.002 |
| Ascomycota *Aspergillus flavus*_F3 | 1.63^a^ | 2.48^de^ | 2.06^ae^ | 4.65^cd^ | 8.16^c^ | 0.85^b^ | 4.28^de^ | <0.001 | <0.001 |
| Basidiomycota *Trichosporon asahii* _F4 | 0.07^ac^ | 0.15^ab^ | 0.79^b^ | 0.02^c^ | 0.41^ab^ | 0.37^b^ | 0.03^c^ | 0.001 | 0.001 |
| Ascomycota *Aspergillus amstelodami*_F5 | 0.58^ac^ | 1.53^b^ | 0.67^c^ | 0.65^ad^ | 0.74^d^ | 0.56^acd^ | 0.66^acd^ | 0.003 | 0.009 |
| Ascomycota *Aspergillus flavus*_F6 | 0.29^ad^ | 0.83^b^ | 0.91^abd^ | 1.51^c^ | 1.56^c^ | 0.22^d^ | 1.10^abc^ | <0.001 | <0.001 |
| Ascomycota *Meyerozyma caribbica*_F7 | 0.43^ad^ | 0.25^a^ | 0.80^abc^ | 1.56^b^ | 0.68^bc^ | 0.42^a^ | 0.54^cd^ | 0.003 | 0.008 |
| Ascomycota *Talaromyces_*unidentified_F8 | 0.05^a^ | 0.10^b^ | 0.05^a^ | 0.30^b^ | 0.18^b^ | 0.22^b^ | 0.27^b^ | <0.001 | <0.001 |
| Basidiomycota *Trichosporon asteroides*_F9 | 0.03 | 0.05 | 0.54 | 0.02 | 0.01 | 0.02 | 0.02 | 0.085 | 0.143 |
| Ascomycota *Alternaria_*unidentified_F10 | 0.04^a^ | 0.18^b^ | 10.96^b^ | 0.09^a^ | 0.00^c^ | 0.01^c^ | 0.05^ac^ | <0.001 | <0.001 |
| Ascomycota *Scopulariopsis brevicaulis*_F11 | 0.17 | 1.96 | 1.44 | 1.40 | 2.76 | 2.18 | 1.05 | 0.047 | 0.087 |
| Ascomycota *Saccharomycopsis fibuligera*_F12 | 0.00^a^ | 0.00^a^ | 0.09^b^ | 1.40^c^ | 0.14^d^ | 0.00^a^ | 0.00^a^ | <0.001 | <0.001 |
| Zygomycota *Rhizopus oryzae*_F13 | 0.18^a^ | 0.50^b^ | 0.62^ab^ | 0.51^b^ | 1.22^c^ | 0.04^d^ | 0.24^ab^ | <0.001 | <0.001 |
| Ascomycota *Davidiella_*unidentified_F14 | 0.00^a^ | 0.07^bc^ | 6.15^b^ | 2.97^b^ | 0.00^ac^ | 0.00^a^ | 0.00^a^ | <0.001 | <0.001 |
| Ascomycota *Davidiella_*unidentified_F15 | 0.00^ab^ | 0.00^a^ | 0.19^cd^ | 0.13^c^ | 0.00^bcd^ | 0.84^c^ | 0.07^ad^ | <0.001 | <0.001 |
| Ascomycota *Fusarium cf equiseti* MY_2011_F16 | 0.70^a^ | 1.45^b^ | 0.30^ce^ | 0.11^ce^ | 0.04^d^ | 0.12^cd^ | 0.17^e^ | <0.001 | <0.001 |
| Ascomycota *Stenocarpella maydis*_F17 | 2.63^a^ | 5.21^b^ | 0.32^c^ | 0.35^c^ | 0.19^d^ | 0.52^c^ | 0.35^c^ | <0.001 | <0.001 |
| Ascomycota *Hypocreales_*unidentified*_*F18 | 0.00^a^ | 0.00^a^ | 0.01^b^ | 0.00^a^ | 0.00^a^ | 0.00^a^ | 0.00^a^ | 0.001 | 0.004 |
| Ascomycota *Acremonium strictum*_F19 | 0.43^a^ | 0.61^b^ | 0.27^cd^ | 0.37^ac^ | 0.25^cd^ | 0.18^d^ | 0.19^d^ | <0.001 | <0.001 |
| Ascomycota *Aureobasidium pullulans*_F20 | 0.00^a^ | 0.00^a^ | 3.77^b^ | 0.12^b^ | 0.00^a^ | 0.01^a^ | 0.04^a^ | <0.001 | <0.001 |
| Ascomycota *Kabatiella_*unidentified_F21 | 0.00^a^ | 0.00^a^ | 6.42^b^ | 0.00^c^ | 0.00^a^ | 0.00^a^ | 0.00^a^ | <0.001 | <0.001 |
| Ascomycota *Pichia_*unidentified_F22 | 0.05^a^ | 0.04^a^ | 0.10^ac^ | 1.05^bc^ | 0.12^ad^ | 0.59^b^ | 0.30^bcd^ | 0.001 | 0.002 |
| Ascomycota *Aspergillus awamori_*F23 | 0.06^a^ | 0.16^ab^ | 0.36^b^ | 0.47^c^ | 0.26^bc^ | 0.13^ad^ | 0.25^bcd^ | <0.001 | <0.001 |
| Ascomycota *Phialemonium curvatum_*F24 | 0.13 | 0.07 | 0.17 | 0.85 | 0.10 | 0.14 | 0.31 | 0.395 | 0.487 |
| Ascomycota *Fusarium annulatum_*F25 | 0.87^a^ | 2.10^b^ | 0.11^cd^ | 0.15^c^ | 0.11^d^ | 0.15^cd^ | 0.30^e^ | <0.001 | <0.001 |

**Note:** Mean relative abundances (%) of the 25 most abundant fungal ASVs are shown, with 12 samples per intestinal segment. Statistical significance was determined using non-parametric Kruskal-Wallis test and *P*-values were further corrected for multiple comparisons using the Benjamini-Hochberg correction. For columns with an FDR ≤ 0.05, pairwise comparisons were performed with Mann-Whitney U test. The values in a row not sharing a common superscript are considered significantly different (*P* < 0.05).

**Supplementary Table S4: Pairwise ANOSIM of β-diversity of the chicken intestinal mycobiota during development**

|  | D3 | D7 | D14 | D21 | D27 | D35 | D42 |
| --- | --- | --- | --- | --- | --- | --- | --- |
| Duodenum |  |  |  |  |  |  |  |
| D3 |  | *< 0.001 (0.279)* | *0.031 (0.121)* | *< 0.001 (0.604)* | *< 0.001 (0.687)* | *< 0.001 (0.811)* | *< 0.001 (0.587)* |
| D7 | 0.174 (0.040) |  | *0.009 (0.190)* | *< 0.001 (0.883)* | *< 0.001 (0.928)* | *< 0.001 (0.999)* | *< 0.001 (0.884)* |
| D14 | 0.407 (0.003) |  |  | *< 0.001 (0.838)* | *< 0.001 (0.910)* | *< 0.001 (0.999)* | *0.001 (0.919)* |
| D21 | 0.005 (0.218) | 0.002 (0.280) | 0.365 (0.014) |  | *< 0.001 (0.792)* | *< 0.001 (0.965)* | *< 0.001 (0.804)* |
| D27 | 0.006 (0.233) | < 0.001 (0.344) | 0.105 (0.076) | 0.060 (0.121) |  | *0.641 (0.024)* | *0.262 (0.031)* |
| D35 | 0.015 (0.149) | < 0.001 (0.302) | 0.004 (0.221) | < 0.001 (0.330) | 0.011 (0.163) |  | *0.505 (0.016)* |
| D42 | 0.047 (0.103) | 0.001 (0.270) | 0.009 (0.152) | < 0.001 (0.331) | 0.066 (0.081) | 0.651 (0.022) |  |
| Jejunum |  |  |  |  |  |  |  |
| D3 |  | *0.002 (0.230)* | *< 0.001 (0.814)* | *< 0.001 (0.678)* | *< 0.001 (0.762)* | *< 0.001 (0.788)* | *< 0.001 (0.816)* |
| D7 | 0.014 (0.097) |  | *0.001 (0.241)* | *0.002 (0.202)* | *< 0.001 (0.353)* | *< 0.001 (0.365)* | *< 0.001 (0.653)* |
| D14 | 0.004 (0.167) | 0.001 (0.246) |  | *0.006 (0.136)* | *< 0.001 (0.364)* | *< 0.001 (0.437)* | *< 0.001 (0.865)* |
| D21 | < 0.001 (0.261) | < 0.001 (0.414) | 0.544 (0.015) |  | *0.144 (0.056)* | *0.013 (0.132)* | *< 0.001 (0.835)* |
| D27 | < 0.001 (0.470) | < 0.001 (0.599) | 0.013 (0.200) | 0.007 (0.214) |  | *0.397 (0.011)* | *< 0.001 (0.861)* |
| D35 | 0.010 (0.123) | 0.016 (0.128) | 0.312 (0.009) | 0.105 (0.062) | < 0.001 (0.374) |  | *< 0.001 (0.913)* |
| D42 | 0.198 (0.021) | 0.055 (0.054) | 0.003 (0.163) | < 0.001 (0.280) | < 0.001 (0.489) | 0.012 (0.097) |  |
| Ileum |  |  |  |  |  |  |  |
| D3 |  | *< 0.001 (0.570)* | *< 0.001 (0.795)* | *< 0.001 (0.742)* | *< 0.001 (0.861)* | *< 0.001 (0.658)* | *< 0.001 (0.872)* |
| D7 | 0.308 (0.009) |  | *0.305 (0.027)* | *0.004 (0.156)* | *< 0.001 (0.295)* | *< 0.001 (0.176)* | *< 0.001 (0.411)* |
| D14 | 0.078 (0.055) | 0.318 (0.006) |  | *0.001 (0.158)* | *< 0.001 (0.350)* | *< 0.001 (0.289)* | *< 0.001 (0.564)* |
| D21 | < 0.001 (0.584) | < 0.001 (0.497) | < 0.001 (0.419) |  | *0.126 (0.054)* | *< 0.001 (0.200)* | *< 0.001 (0.436)* |
| D27 | < 0.001 (0.394) | < 0.001 (0.309) | 0.004 (0.212) | 0.101 (0.068) |  | *0.003 (0.125)* | *< 0.001 (0.310)* |
| D35 | < 0.001 (0.316) | < 0.001 (0.254) | 0.011 (0.148) | 0.002 (0.239) | 0.257 (0.018) |  | *0.792 (0.032)* |
| D42 | 0.001 (0.199) | < 0.001 (0.141) | 0.025 (0.095) | < 0.001 (0.404) | 0.003 (0.154) | 0.138 (0.041) |  |
| Cecum |  |  |  |  |  |  |  |
| D3 |  | *0.074 (0.062)* | *0.008 (0.122)* | *0.004 (0.211)* | *< 0.001 (0.230)* | *< 0.001 (0.383)* | *< 0.001 (0.370)* |
| D7 | 0.199 (0.025) |  | *0.015 (0.128)* | *< 0.001 (0.499)* | *< 0.001 (0.507)* | *< 0.001 (0.773)* | *< 0.001 (0.616)* |
| D14 | 0.040 (0.068) |  |  | *0.013 (0.158)* | *0.118 (0.079)* | *< 0.001 (0.721)* | < *0.001 (0.467)* |
| D21 | < 0.001 (0.423) | < 0.001 (0.389) | 0.036 (0.102) |  | *0.137 (0.065)* | *< 0.001 (0.483)* | *0.004 (0.258)* |
| D27 | < 0.001 (0.539) | < 0.001 (0.507) | 0.027 (0.117) | 0.152 (0.039) |  | *< 0.001 (0.426)* | *< 0.001 (0.225)* |
| D35 | 0.007 (0.163) | 0.011 (0.153) | 0.373 (0.003) | 0.005 (0.176) | 0.011 (0.191) |  | *0.019 (0.137)* |
| D42 | < 0.001 (0.193) | < 0.001 (0.185) | 0.073 (0.041) | < 0.001 (0.282) | < 0.001 (0.272) | 0.806 (0.039) |  |

**Note:** P and R values of pairwise comparisons based on weighted and unweighted UniFrac distances were determined by ANOSIM with 999 permutations. R value for each comparison is shown in parenthesis. Those values for unweighted UniFrac are italicized.

**Supplementary Table S5: Temporal shifts in the chicken mycobiota composition (%)**

|  | D3 | D7 | D14 | D21 | D27 | D35 | D42 | P-Value | FDR |
| --- | --- | --- | --- | --- | --- | --- | --- | --- | --- |
| Duodenum |  |  |  |  |  |  |  |  |  |
| Ascomycota *Fusarium pseudonygamai*_F1 | 50.56 | 44.97 | 34.46 | 25.98 | 38.02 | 56.29 | 52.44 | 0.101 | 0.224 |
| Ascomycota *Candida albicans­_*F2 | 0.72^a^ | 1.88^ab^ | 4.25^ac^ | 12.50^d^ | 8.08^cd^ | 13.96^cd^ | 2.14^bc^ | <0.001 | 0.002 |
| Ascomycota *Aspergillus flavus*_F3 | 0.75^ad^ | 3.55^b^ | 1.85^acd^ | 0.63^d^ | 1.34^ade^ | 3.30^abe^ | 2.06^bce^ | 0.001 | 0.006 |
| Basidiomycota *Trichosporon asahii*_F4 | 0.59^ac^ | 2.15^ac^ | 10.01^a^ | 10.87^abc^ | 12.38^b^ | 0.59^c^ | 0.79^ac^ | 0.002 | 0.007 |
| Ascomycota *Aspergillus amstelodami*_F5 | 0.39^ac^ | 5.65^a^ | 2.06^ab^ | 1.63^ab^ | 0.69^c^ | 0.14^c^ | 0.67^bc^ | 0.002 | 0.009 |
| Ascomycota *Aspergillus flavus*_F6 | 0.64 | 1.36 | 0.72 | 0.23 | 1.29 | 0.56 | 0.91 | 0.509 | 0.589 |
| Ascomycota *Meyerozyma caribbica*_F7 | 1.30^ab^ | 0.05^a^ | 0.38^ab^ | 0.44^bc^ | 0.96^b^ | 0.10^ac^ | 0.80^b^ | 0.006 | 0.019 |
| Ascomycota *Talaromyces_*unidentified_F8 | 0.14^a^ | 0.52^a^ | 0.08^a^ | 0.00^b^ | 0.16^a^ | 0.16^a^ | 0.05^a^ | 0.003 | 0.011 |
| Basidiomycota *Trichosporon asteroides*_F9 | 0.22 | 0.07 | 0.779 | 4.26 | 4.81 | 0.23 | 0.54 | 0.030 | 0.082 |
| Ascomycota *Alternaria_*unidentified_F10 | 0.07^ad^ | 0.12^bd^ | 0.00^a^ | 0.44^abc^ | 3.36^bc^ | 0.22^bc^ | 10.96^c^ | <0.001 | 0.001 |
| Ascomycota *Scopulariopsis brevicaulis*_F11 | 0.00^ab^ | 0.00^a^ | 0.00^a^ | 0.03^b^ | 4.59^c^ | 0.50^d^ | 1.44^cd^ | <0.001 | <0.001 |
| Ascomycota *Saccharomycopsis fibuligera*_F12 | 7.93^a^ | 9.92^a^ | 9.14^a^ | 16.24^a^ | 2.60^b^ | 0.01^b^ | 0.09^b^ | <0.001 | <0.001 |
| Zygomycota *Rhizopus oryzae*_F13 | 0.58^bc^ | 1.38^b^ | 0.86^ab^ | 1.43^b^ | 0.30^ac^ | 0.17^a^ | 0.62^ab^ | 0.001 | 0.006 |
| Ascomycota *Davidiella_*unidentified_F14 | 9.08 | 0.71 | 1.98 | 0.23 | 0.31 | 0.31 | 6.15 | 0.061 | 0.152 |
| Ascomycota *Davidiella_*unidentified_F15 | 0.05^cd^ | 0.86^cd^ | 0.15^c^ | 1.99^ad^ | 0.39^ab^ | 6.74^b^ | 0.19^ac^ | <0.001 | 0.001 |
| Ascomycota *Fusarium cf equiseti* MY_2011_F16 | 3.49^cd^ | 5.02^c^ | 2.26^c^ | 3.39^c^ | 0.95^b^ | 0.87^ad^ | 0.30^a^ | 0.001 | 0.003 |
| Ascomycota *Stenocarpella maydis*_F17 | 0.40a | 0.10^a^ | 0.49^ad^ | 0.03^b^ | 0.59^cd^ | 0.25^cd^ | 0.32^c^ | <0.001 | <0.001 |
| Ascomycota *Hypocreales_*unidentified*_*F18 | 7.11^b^ | 9.30^b^ | 8.48^b^ | 7.62^b^ | 1.85^a^ | 0.04^a^ | 0.01^a^ | <0.001 | <0.001 |
| Ascomycota *Acremonium strictum*_F19 | 1.06^abc^ | 0.08^ab^ | 0.03^a^ | 0.09^ab^ | 0.11^bd^ | 0.38^c^ | 0.27^cd^ | 0.001 | 0.003 |
| Ascomycota *Aureobasidium pullulans*_F20 | 0.09^ad^ | 0.02^a^ | 0.03^a^ | 1.59^b^ | 0.47^bc^ | 1.76^c^ | 3.77^bcd^ | <0.001 | <0.001 |
| Ascomycota *Kabatiella_*unidentified_F21 | 0.00^a^ | 0.00^a^ | 0.00^a^ | 0.00^a^ | 0.55^b^ | 0.74^b^ | 6.42^b^ | <0.001 | <0.001 |
| Ascomycota *Pichia_*unidentified_F22 | 0.23 | 0.02 | 0.80 | 0.67 | 0.58 | 0.23 | 0.10 | 0.120 | 0.259 |
| Ascomycota *Aspergillus awamori_*F23 | 0.83 | 0.41 | 0.62 | 0.23 | 0.23 | 0.36 | 0.36 | 0.409 | 0.566 |
| Ascomycota *Phialemonium curvatum_*F24 | 0.02^ab^ | 0.03^a^ | 0.00^b^ | 0.02^ab^ | 0.12^ac^ | 0.21^c^ | 0.17^c^ | <0.001 | 0.001 |
| Ascomycota *Fusarium annulatum_*F25 | 3.39 | 0.06 | 0.05 | 0.19 | 0.03 | 0.03 | 0.11 | 0.038 | 0.099 |
| Jejunum |  |  |  |  |  |  |  |  |  |
| Ascomycota *Fusarium pseudonygamai*_F1 | 71.78^ab^ | 78.36^a^ | 68.41^b^ | 68.66^b^ | 54.51^c^ | 73.71^ab^ | 66.70^abc^ | 0.004 | 0.005 |
| Ascomycota *Candida albicans­_*F2 | 1.23^a^ | 2.03^a^ | 9.80^b^ | 13.76^b^ | 11.73^b^ | 11.32^b^ | 1.74^c^ | <0.001 | <0.001 |
| Ascomycota *Aspergillus flavus*_F3 | 4.69^ad^ | 5.45^b^ | 3.84^ad^ | 1.50^c^ | 3.06^d^ | 4.76^ab^ | 4.65^abd^ | <0.001 | <0.001 |
| Basidiomycota *Trichosporon asahii*_F4 | 0.24^ad^ | 0.16^ad^ | 2.17^bc^ | 3.13^bc^ | 9.14^b^ | 0.84^ac^ | 0.02^d^ | <0.001 | <0.001 |
| Ascomycota *Aspergillus amstelodami*_F5 | 0.54^a^ | 0.94^b^ | 2.48^c^ | 3.58^c^ | 3.59^c^ | 0.70^ab^ | 0.65^a^ | <0.001 | <0.001 |
| Ascomycota *Aspergillus flavus*_F6 | 2.75^a^ | 2.78^b^ | 1.96^a^ | 0.64^c^ | 1.43^a^ | 1.28^a^ | 1.51^a^ | <0.001 | <0.001 |
| Ascomycota *Meyerozyma caribbica*_F7 | 5.06 | 1.75 | 1.78 | 1.48 | 2.05 | 1.32 | 1.56 | 0.632 | 0.658 |
| Ascomycota *Talaromyces_*unidentified_F8 | 0.33^ac^ | 0.34^b^ | 0.26^c^ | 0.12^a^ | 0.18^a^ | 0.17^a^ | 0.30^ac^ | 0.001 | 0.001 |
| Basidiomycota *Trichosporon asteroides*_F9 | 0.03^a^ | 0.12^ab^ | 3.08^c^ | 1.76^c^ | 4.84^bc^ | 0.41^bc^ | 0.02^a^ | <0.001 | <0.001 |
| Ascomycota *Alternaria_*unidentified_F10 | 1.03^a^ | 0.33^b^ | 0.03^cd^ | 0.09^cd^ | 0.01^c^ | 0.11^cd^ | 0.00^d^ | <0.001 | <0.001 |
| Ascomycota *Scopulariopsis brevicaulis*_F11 | 0.05^a^ | 0.03^a^ | 0.12^b^ | 0.19^b^ | 3.70^c^ | 0.53^d^ | 1.40^d^ | <0.001 | <0.001 |
| Ascomycota *Saccharomycopsis fibuligera*_F12 | 0.00^a^ | 0.00^a^ | 0.00^a^ | 0.00^a^ | 0.00^a^ | 0.00^a^ | 1.40^b^ | <0.001 | <0.001 |
| Zygomycota *Rhizopus oryzae*_F13 | 0.31 | 0.55 | 0.74 | 0.56 | 0.52 | 0.50 | 0.51 | 0.082 | 0.093 |
| Ascomycota *Davidiella_*unidentified_F14 | 0.00^ab^ | 0.15^ab^ | 0.00^a^ | 0.63^bcd^ | 0.04^c^ | 0.02^d^ | 2.97^e^ | <0.001 | <0.001 |
| Ascomycota *Davidiella_*unidentified_F15 | 0.15^a^ | 0.00^b^ | 0.00^bc^ | 0.02^ac^ | 0.00^b^ | 0.00^b^ | 0.13^a^ | <0.001 | <0.001 |
| Ascomycota *Fusarium cf equiseti* MY_2011_F16 | 2.66^a^ | 0.32^a^ | 0.39^b^ | 0.11^b^ | 0.12^b^ | 0.13^b^ | 0.11^b^ | <0.001 | <0.001 |
| Ascomycota *Stenocarpella maydis*_F17 | 0.56^a^ | 0.86^ab^ | 0.45^ab^ | 0.20^b^ | 1.65^c^ | 0.47^ab^ | 0.35^a^ | 0.001 | 0.001 |
| Ascomycota *Hypocreales_*unidentified*_*F18 | 0.00 | 0.00 | 0.00 | 0.00 | 0.00 | 0.00 | 0.00 | 0.760 | 0.760 |
| Ascomycota *Acremonium strictum*_F19 | 0.39^ab^ | 0.75^a^ | 0.66^c^ | 0.31^ab^ | 0.23^b^ | 0.45^ac^ | 0.37^ac^ | 0.009 | 0.012 |
| Ascomycota *Aureobasidium pullulans*_F20 | 0.02^a^ | 0.17^a^ | 0.00^a^ | 0.00^a^ | 0.00^a^ | 0.00^a^ | 0.12^b^ | <0.001 | <0.001 |
| Ascomycota *Kabatiella_*unidentified_F21 | 0.01^ab^ | 0.28^ac^ | 0.00^c^ | 0.00^c^ | 0.00^c^ | 0.00^c^ | 0.00^b^ | <0.001 | <0.001 |
| Ascomycota *Pichia_*unidentified_F22 | 0.04^a^ | 0.49^ab^ | 0.02^c^ | 0.05^bc^ | 0.03^ac^ | 0.14^a^ | 1.05^d^ | <0.001 | <0.001 |
| Ascomycota *Aspergillus awamori_*F23 | 0.80^a^ | 0.47^a^ | 0.24^ab^ | 0.09^c^ | 0.17^b^ | 0.15^b^ | 0.47^a^ | <0.001 | <0.001 |
| Ascomycota *Phialemonium curvatum_*F24 | 0.05^a^ | 0.16^b^ | 0.17^b^ | 0.05^a^ | 0.08^ab^ | 0.07^ab^ | 0.85^ab^ | 0.016 | 0.018 |
| Ascomycota *Fusarium annulatum_*F25 | 0.17 | 0.15 | 0.15 | 0.25 | 0.34 | 0.43 | 0.15 | 0.110 | 0.120 |
| Ileum |  |  |  |  |  |  |  |  |  |
| Ascomycota *Fusarium pseudonygamai*_F1 | 78.32^a^ | 74.46^a^ | 76.47^a^ | 55.76^b^ | 65.95^b^ | 66.98^b^ | 74.66^b^ | <0.001 | 0.001 |
| Ascomycota *Candida albicans­_*F2 | 1.09^a^ | 1.20^a^ | 4.60^ad^ | 16.91^b^ | 11.31^bc^ | 10.75^c^ | 6.29^cd^ | <0.001 | <0.001 |
| Ascomycota *Aspergillus flavus*_F3 | 6.22^ad^ | 4.22^ad^ | 5.61^ad^ | 3.61^a^ | 5.66^ab^ | 8.78^b^ | 8.16^bd^ | 0.006 | 0.009 |
| Basidiomycota *Trichosporon asahii* _F4 | 0.11^a^ | 0.30^b^ | 1.04^b^ | 3.60^c^ | 4.37^c^ | 0.24^ab^ | 0.41^b^ | <0.001 | <0.001 |
| Ascomycota *Aspergillus amstelodami*_F5 | 1.02^ab^ | 1.73^a^ | 2.50^c^ | 9.41^d^ | 2.36^c^ | 1.51^ac^ | 0.74^b^ | <0.001 | <0.001 |
| Ascomycota *Aspergillus flavus*_F6 | 3.49 | 2.23 | 2.82 | 1.96 | 2.66 | 3.14 | 1.56 | 0.123 | 0.128 |
| Ascomycota *Meyerozyma caribbica*_F7 | 0.85^a^ | 0.64^ab^ | 0.32^b^ | 0.66^a^ | 0.60^a^ | 0.70^a^ | 0.68^a^ | 0.018 | 0.023 |
| Ascomycota *Talaromyces_*unidentified_F8 | 0.45^a^ | 6.14^b^ | 0.64^b^ | 0.15^c^ | 0.25^ad^ | 0.29^a^ | 0.18^cd^ | <0.001 | <0.001 |
| Basidiomycota *Trichosporon asteroides*_F9 | 0.01^a^ | 0.01^a^ | 0.09^a^ | 2.82^b^ | 0.40^b^ | 0.29^a^ | 0.01^a^ | <0.001 | <0.001 |
| Ascomycota *Alternaria_*unidentified_F10 | 0.03^a^ | 1.06^b^ | 0.14c | 0.01^ad^ | 0.00^e^ | 0.00^de^ | 0.00^de^ | <0.001 | <0.001 |
| Ascomycota *Scopulariopsis brevicaulis*_F11 | 0.07^a^ | 0.10^ab^ | 0.02^b^ | 0.78^c^ | 2.16^c^ | 2.27^c^ | 2.76^c^ | <0.001 | <0.001 |
| Ascomycota *Saccharomycopsis fibuligera*_F12 | 0.08^a^ | 0.08^be^ | 0.00^c^ | 0.00^c^ | 0.03^d^ | 0.03^b^ | 0.14^ae^ | <0.001 | <0.001 |
| Zygomycota *Rhizopus oryzae*_F13 | 0.48^a^ | 0.89^b^ | 0.84^ab^ | 1.06^b^ | 0.91^ab^ | 1.41^b^ | 1.22^b^ | 0.025 | 0.030 |
| Ascomycota *Davidiella_*unidentified_F14 | 0.13^a^ | 0.12^bc^ | 0.00^bd^ | 0.01^c^ | 0.00^d^ | 0.03^bc^ | 0.00^c^ | <0.001 | 0.001 |
| Ascomycota *Davidiella_*unidentified_F15 | 0.00 | 0.01 | 0.00 | 0.01 | 0.00 | 0.00 | 0.00 | 0.081 | 0.089 |
| Ascomycota *Fusarium cf equiseti* MY_2011_F16 | 0.15^a^ | 0.17^a^ | 0.22^a^ | 0.08^b^ | 0.05^b^ | 0.06^b^ | 0.04^b^ | <0.001 | <0.001 |
| Ascomycota *Stenocarpella maydis*_F17 | 0.55 | 0.20 | 0.37 | 0.21 | 0.16 | 0.40 | 0.19 | 0.080 | 0.089 |
| Ascomycota *Hypocreales_*unidentified*_*F18 | 0.00^a^ | 0.00^b^ | 0.00^b^ | 0.00^ab^ | 0.00^b^ | 0.00^b^ | 0.00^ab^ | 0.017 | 0.022 |
| Ascomycota *Acremonium strictum*_F19 | 0.59^ac^ | 0.32^ab^ | 0.47^a^ | 0.20^b^ | 0.24^bc^ | 0.36^ab^ | 0.25^b^ | 0.014 | 0.019 |
| Ascomycota *Aureobasidium pullulans*_F20 | 0.96^a^ | 0.00^b^ | 0.05^b^ | 0.00^b^ | 0.00^b^ | 0.00^b^ | 0.00^b^ | <0.001 | <0.001 |
| Ascomycota *Kabatiella_*unidentified_F21 | 0.06^a^ | 0.00^b^ | 0.00^b^ | 0.00^b^ | 0.00^b^ | 0.00^b^ | 0.00^b^ | <0.001 | <0.001 |
| Ascomycota *Pichia_*unidentified_F22 | 0.10^a^ | 0.28^ab^ | 0.03^b^ | 0.03^b^ | 0.03^b^ | 0.05^bc^ | 0.12^ac^ | 0.010 | 0.015 |
| Ascomycota *Aspergillus awamori_*F23 | 0.79^a^ | 0.47^abc^ | 0.62^a^ | 0.21^b^ | 0.31^bc^ | 0.36^c^ | 0.26^bc^ | 0.001 | 0.001 |
| Ascomycota *Phialemonium curvatum_*F24 | 0.09^acd^ | 2.51^b^ | 0.39^b^ | 0.02^a^ | 0.04^ac^ | 0.06^cd^ | 0.10^d^ | <0.001 | <0.001 |
| Ascomycota *Fusarium annulatum_*F25 | 0.14 | 0.17 | 0.15 | 0.14 | 0.11 | 0.12 | 0.11 | 0.493 | 0.493 |
| Cecum |  |  |  |  |  |  |  |  |  |
| Ascomycota *Fusarium pseudonygamai*_F1 | 91.73^ad^ | 89.33^ac^ | 81.02^abc^ | 66.42^b^ | 78.45^bc^ | 84.51^cd^ | 84.88^c^ | 0.001 | 0.001 |
| Ascomycota *Candida albicans­_*F2 | 0.15^a^ | 0.81^b^ | 2.51^bc^ | 9.19^cd^ | 8.70^d^ | 7.42^d^ | 5.21^cd^ | <0.001 | <0.001 |
| Ascomycota *Aspergillus flavus*_F3 | 0.21^a^ | 0.45^a^ | 0.42^ab^ | 0.58^abc^ | 0.42^abc^ | 0.73^bc^ | 0.85c | 0.005 | 0.009 |
| Basidiomycota *Trichosporon asahii* _F4 | 0.14^a^ | 0.13^b^ | 1.75^c^ | 3.51^d^ | 2.73^d^ | 0.19^bc^ | 0.37^bc^ | <0.001 | <0.001 |
| Ascomycota *Aspergillus amstelodami*_F5 | 0.05^a^ | 0.22^ab^ | 0.22^bc^ | 1.51^d^ | 0.32^cd^ | 0.45^d^ | 0.56^d^ | <0.001 | <0.001 |
| Ascomycota *Aspergillus flavus*_F6 | 0.09^ab^ | 0.05^a^ | 0.28^b^ | 0.21^b^ | 0.13^b^ | 0.26^b^ | 0.22^b^ | 0.029 | 0.040 |
| Ascomycota *Meyerozyma caribbica*_F7 | 0.23 | 0.32 | 0.32 | 0.59 | 0.39 | 0.29 | 0.42 | 0.653 | 0.700 |
| Ascomycota *Talaromyces_*unidentified_F8 | 0.10^ab^ | 0.49^a^ | 0.42^abc^ | 7.63^c^ | 0.31^cd^ | 0.15^bd^ | 0.22^bc^ | 0.004 | 0.007 |
| Basidiomycota *Trichosporon asteroides*_F9 | 0.08^a^ | 0.06^a^ | 2.49^b^ | 6.64^c^ | 1.32^bc^ | 0.01^a^ | 0.02^a^ | <0.001 | <0.001 |
| Ascomycota *Alternaria_*unidentified_F10 | 0.66^ab^ | 0.18^ab^ | 3.12^a^ | 0.30^c^ | 0.18^a^ | 0.00^d^ | 0.01^bd^ | <0.001 | <0.001 |
| Ascomycota *Scopulariopsis brevicaulis*_F11 | 0.02^a^ | 0.01^ab^ | 0.03^b^ | 0.29^c^ | 1.03^c^ | 0.84^c^ | 2.18^c^ | <0.001 | <0.001 |
| Ascomycota *Saccharomycopsis fibuligera*_F12 | 0.00 | 0.00 | 0.00 | 0.00 | 0.00 | 0.00 | 0.00 | 0.034 | 0.045 |
| Zygomycota *Rhizopus oryzae*_F13 | 0.01^a^ | 0.00^a^ | 0.01^a^ | 0.01^ab^ | 0.00^a^ | 0.02^bc^ | 0.04^c^ | <0.001 | <0.001 |
| Ascomycota *Davidiella_*unidentified_F14 | 0.05 | 0.00 | 0.00 | 0.00 | 0.00 | 0.00 | 0.00 | 0.072 | 0.090 |
| Ascomycota *Davidiella_*unidentified_F15 | 0.01^ab^ | 0.18^b^ | 0.01^a^ | 0.03^a^ | 0.01^ab^ | 0.03^c^ | 0.84^c^ | <0.001 | <0.001 |
| Ascomycota *Fusarium cf equiseti* MY_2011_F16 | 0.03^ac^ | 0.02^a^ | 0.02^a^ | 0.02^ac^ | 0.05^ab^ | 0.10^b^ | 0.12^bc^ | 0.015 | 0.024 |
| Ascomycota *Stenocarpella maydis*_F17 | 0.86 | 0.37 | 0.45 | 0.40 | 0.45 | 0.59 | 0.52 | 0.290 | 0.345 |
| Ascomycota *Hypocreales_*unidentified*_*F18 | 0.00 | 0.12 | 0.00 | 0.00 | 0.00 | 0.00 | 0.00 | 0.682 | 0.700 |
| Ascomycota *Acremonium strictum*_F19 | 0.92^a^ | 0.81^a^ | 0.96^ad^ | 0.43^b^ | 0.60^ab^ | 0.50^bd^ | 0.18^c^ | <0.001 | <0.001 |
| Ascomycota *Aureobasidium pullulans*_F20 | 0.22^ab^ | 0.56^b^ | 3.07^a^ | 0.01^cd^ | 0.31^ad^ | 0.00^c^ | 0.01^cd^ | <0.001 | <0.001 |
| Ascomycota *Kabatiella_*unidentified_F21 | 0.00 | 0.00 | 0.00 | 0.00 | 0.00 | 0.00 | 0.00 | 0.481 | 0.547 |
| Ascomycota *Pichia_*unidentified_F22 | 0.98 | 1.67 | 0.58 | 0.43 | 0.61 | 0.32 | 0.59 | 0.700 | 0.700 |
| Ascomycota *Aspergillus awamori_*F23 | 0.07^a^ | 0.25^a^ | 0.06^ab^ | 0.02^a^ | 0.04^ac^ | 0.11^bc^ | 0.13^b^ | 0.005 | 0.009 |
| Ascomycota *Phialemonium curvatum_*F24 | 0.23^abc^ | 0.41^a^ | 0.36^ac^ | 0.22^c^ | 0.15^bc^ | 0.23^ac^ | 0.14^b^ | 0.019 | 0.028 |
| Ascomycota *Fusarium annulatum_*F25 | 0.09^bc^ | 0.08^bc^ | 0.08^bc^ | 0.06^b^ | 0.14^ac^ | 0.17^a^ | 0.15^ab^ | 0.002 | 0.003 |

**Note:** Mean relative abundances (%) of the 25 most abundant fungal ASVs are shown, with 12 samples per intestinal segment. Statistical significance was determined using non-parametric Kruskal-Wallis test and *P*-values were further corrected for multiple comparisons using the Benjamini-Hochberg correction. For columns with an FDR ≤ 0.05, pairwise comparisons were performed with Mann-Whitney U test. The values in a row not sharing a common superscript are considered significantly different (*P* < 0.05).
